# Foundations for Streaming Model Transformations by Complex Event Processing

**DOI:** 10.1007/s10270-016-0533-1

**Published:** 2016-05-26

**Authors:** István Dávid, István Ráth, Dániel Varró

**Affiliations:** 1 Department of Mathematics and Computer Science, University of Antwerp, Middelheimlaan 1, 2020 Antwerp, Belgium; 2 Department of Measurement and Information Systems, Budapest University of Technology and Economics, Magyar tudósok krt. 2., Budapest, 1117 Hungary; 3 IncQuery Labs Ltd., Bocskai út 77-79, Budapest, 1113 Hungary; 4 MTA-BME Lendület Research Group on Cyber-Physical Systems, Budapest, Hungary

**Keywords:** Streaming model transformations, Complex event processing, Live models, Change-driven transformations, Reactive transformations

## Abstract

Streaming model transformations represent a novel class of transformations to manipulate models whose elements are continuously produced or modified in high volume and with rapid rate of change. Executing streaming transformations requires efficient techniques to recognize activated transformation rules over a live model and a potentially infinite stream of events. In this paper, we propose foundations of streaming model transformations by innovatively integrating incremental model query, complex event processing (CEP) and reactive (event-driven) transformation techniques. Complex event processing allows to identify relevant patterns and sequences of events over an event stream. Our approach enables event streams to include model change events which are automatically and continuously populated by incremental model queries. Furthermore, a reactive rule engine carries out transformations on identified complex event patterns. We provide an integrated domain-specific language with precise semantics for capturing complex event patterns and streaming transformations together with an execution engine, all of which is now part of the Viatra reactive transformation framework. We demonstrate the feasibility of our approach with two case studies: one in an advanced model engineering workflow; and one in the context of on-the-fly gesture recognition.

## Introduction

*Live models in smart cyber-physical systems* Smart Cyber-Physical Systems [56, 57] are open, interconnected and highly distributed complex systems expected to consist of 50 billion smart objects and devices by 2020 [17], which integrate simple sensors and actuators to the Internet-of-Things (IoT) [73] to exploit the user interface of mobile devices and the computational power of cloud-based infrastructures. In many cases, they also connect traditional critical embedded systems where a failure may result in major financial loss, severe damage or even casualties.

Management of such smart systems frequently necessitates soft real-time processing, and it may rely upon a closed control loop which observes data reported by sensors of the system, and interacts with actuators based upon some control logic. Typical applications following such a scenario include run-time reconfiguration and optimization [19] of the underlying system, knowledge maintenance in online machine learning [38], distributed reasoning [41], etc.

Many distributed systems in IoT implement the control logic over a stream of events which may offer extreme scalability in a distributed environment with a massive number of nodes. Complex event processing (CEP) [37, 58] offer well-founded techniques to capture critical *event sequences* observed on the event streams within a given time window which require immediate reaction. The event stream is considered as an external component for the CEP engine, which is *loosely connected to the event sources*, thus adapting a CEP engine to consume model changes as events require significant manual programming effort [62].

However, a smart CPS also needs to autonomously perceive its operational context and adapt to changes in an open, heterogeneous and distributed environment. For that purpose, the current snapshot of the system and its operational context can be formally captured as a *live model* (also referred as models@runtime [15]) which continuously gets updated to reflect relevant changes in the underlying real system. Furthermore, operations executed on this live model may have immediate and direct effect on the running system.

*Toward streaming transformations over live models* Scalability of models, queries and transformations has become a key challenge in model-driven engineering [55] to handle complex scenarios of industrial domains of critical embedded systems like automotive or avionics. Efficient *graph reasoning* [40] techniques (based on constraint or query languages [61, 70, 74]) assist in identifying critical model changes while reactions are regularly defined by rule-based techniques (such as graph transformation [12]). However, the same techniques fail to identify complex *sequences* of model changes.

The maintenance and manipulation of large models also initiated to come up with novel classes of model transformations. *Change-driven transformations* [14] consume or produce changes of source and target models as their input or output models, to enable transformations over partially materialized models and to reduce the amount of traceability information required to be stored in the model. Sánchez Cuadrado and de Lara define *streaming transformations* as a “special kind of transformation in which the whole input model is not completely available at the beginning of the transformation, but it is continuously generated“ [65]. An additional class of streaming transformations aims to tackle very large models by feeding a transformation process incrementally (keeping only a part of the model in memory at any time).

However, in the context of smart CPS, live models may evolve at a very fast rate, or they may not be fully materialized, i.e., only a part of the live model is stored in memory while changes in other component are reported as events. For example, the optical sensors of a CPS may search for a specific pattern over a continuous stream of images, or a runtime monitor (with small memory footprint) may look for a violation of a safety property with temporal constraints. Applying graph reasoning and transformation techniques in the context of live models and IoT applications is still in an early research phase [41, 60].

*Contributions* In [27], we identified a novel class of streaming transformations for live models where the models themselves are not necessarily large or infinite, but they change or evolve at a very fast rate (for instance, 25 times per second), and it is the stream of model changes that requires efficient processing. In this paper, we innovatively combine *complex event processing techniques with live model queries and transformations* where

1. changes of a live model at different (but user-defined) level of granularity can be identified by changes of a query result set and then published as *atomic events* to one or more event streams similarly to external stimuli;
2. relevant event sequences are identified by adapting complex event processing (CEP) techniques [37, 58];
3. transformation rules enable to react to such complex event sequences by manipulating the live models or sending further events.

Our technical contributions include

A a high-level integrated *domain-specific language* for capturing *complex event sequences over model changes defined by queries* and specifying *reactions as streaming transformations*;
B *precise foundations* of this event processing DSL including *syntax and semantics* (both formal algebraic and executable);
C a *complex event processing engine* tightly *integrated into the* Viatra *reactive and incremental transformation framework* [13];
D *initial scalability measurements* to assess the performance of the framework in the context of live models for gesture recognition; and
E a new *case study* of an advanced model-driven engineering tooling workflow in the context of CPS.

While the technical depth of presentation increased in general wrt. the earlier version [27], contributions (B) and (E) are completely novel in the current paper.

The main conceptual added value of our work is the seamless and tight integration between a reactive MT engine and a CEP engine to handle model changes as events and the other way around. As a result, graph reasoning and complex event processing techniques can be simultaneously used in the context of live models without additional programming and integration efforts. Furthermore, introducing compound changes as atomic events significantly reduces the complexity of complex event patterns and their checking automata compared to a solution which relies only on a CEP engine.

*Structure of the paper* In Sect. 2, we give a brief overview on our approach. Section 3 introduces a running example on complex event-driven live model synchronization aided by design space exploration. Section 4 defines the static structure and the formal semantics of the domain-specific language supporting our approach. Section 5 presents the executable semantics of the DSL. In Sect. 6, we elaborate on the case study, using our proposed DSL and architecture. In Sect.7, we present a case study of gesture recognition over live models and carry out the performance evaluation of the approach. Finally, related approaches and tools are described in Sects. 8 and  9 concludes our paper.

## Overview of the Approach

We propose a novel class of streaming model transformations where (1) changes of live models (representing the state of the system) are published as atomic events by an incremental query engine, (2) complex event sequences can be observed over an event stream and (3) reactions to such complex events can be executed by a reactive transformation engine.

**Fig. 1:**
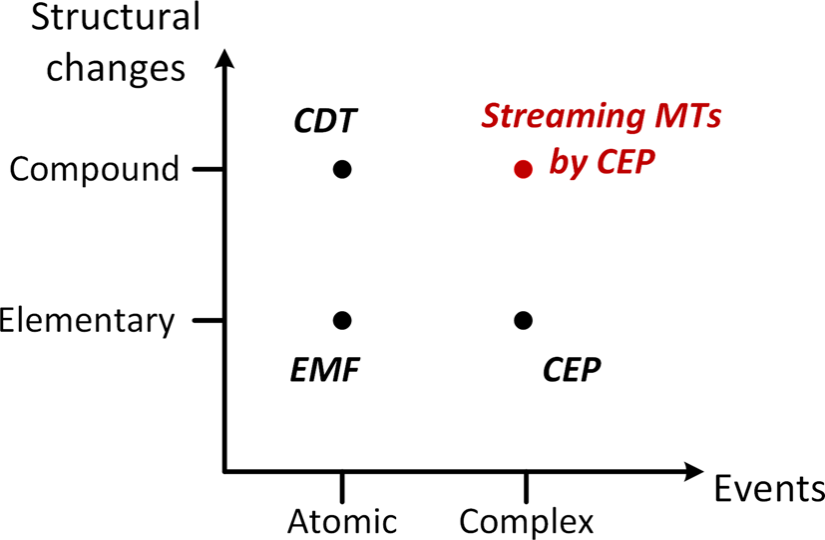
Structural changes versus events

As a terminology, changes affecting the structure of a model (e.g., adding, removing or changing model elements) are called *elementary structural changes*. When relevant elementary changes of the live model are aggregated into a (compound) change which is observed by an appropriate *change pattern*, *change events* are generated in an event stream and offered to a complex event processing engine. Based on the granularity of (i) the observed model changes and (ii) the events mediating that change information to the processing module, we distinguish four main scenarios (see Fig. 1).

### Elementary Structural Changes

In this base case, model changes are elementary modifications (e.g., modifying an attribute of an object, or removing a reference between two objects), while change events can be elementary notifications sent by the model management framework. Notable frameworks supporting elementary structural changes include the Eclipse Modeling Framework (EMF) [32] together with notifiers/adapters via the EMF Notification API or the Kevoree Modeling Framework (KMF) [54].

### From Elementary to Compound Changes

*Compound* structural changes aggregate multiple elementary changes between two states (snapshots) of the model (called the pre-state and the post-state). The techniques of *change-driven transformations* (CDT) [14] were proposed to identify compound structural changes by using *change patterns* [14, 18, 76].

As their main characteristics, change patterns observe the delta between the pre-state and the post-state regardless of the actual trajectory between those states. Thus, if multiple different sequences of elementary changes can lead to the same compound change, CDT is unable to distinguish between those sequences but identify the same compound (aggregate) change.

### From Atomic to Complex Events

To avoid overloading the term “change,” we define an *event (instance)* as a record of significant information on some internal modification in a system or some external change observed in the context of the system at a given point in time (as minor adaption of the definition in [63]).

Concerning the granularity of events, we distinguish between atomic and complex events. *Complex event processing* (CEP) [58] techniques provide solid foundations on how to model and evaluate logical structures of atomic event instances in order to detect (sequences or patterns of) complex events. *Atomic event instance*s can be directly observed on an *event stream*. *Complex event instance*s are constituted from logic structures of multiple atomic event instances, and thus, they cannot be directly observed. Instead, their presence is deduced by processing the atomic event instances.

Complex event processing means matching event instances against previously defined event patterns. *Event patterns* are abstractions of event instances, and they are primarily characterized by the *type* and potentially, some extra parameters. Event instances are further augmented by a *timestamp*, which defines an ordering over relation over a set events to decide in what order events *follow* each other^1^ Additionally, timestamps also enable calculating the length of *time windows* the complex events occur within.

**Table 1:** Advantages and shortcomings of CDT and CEP techniques

	CDT	CEP
Advantages	Efficiently captures change deltas between two states	Efficiently captures sequences
Shortcoming	Fails to distinguish between sequences	Fails to efficiently abstract from actual sequence of events

Complex event patterns are defined using an *event pattern language* and then evaluated using an *event processing algebra* which offers common operators (followed by, and/or, multiplicity, etc.).

**Fig. 2:**
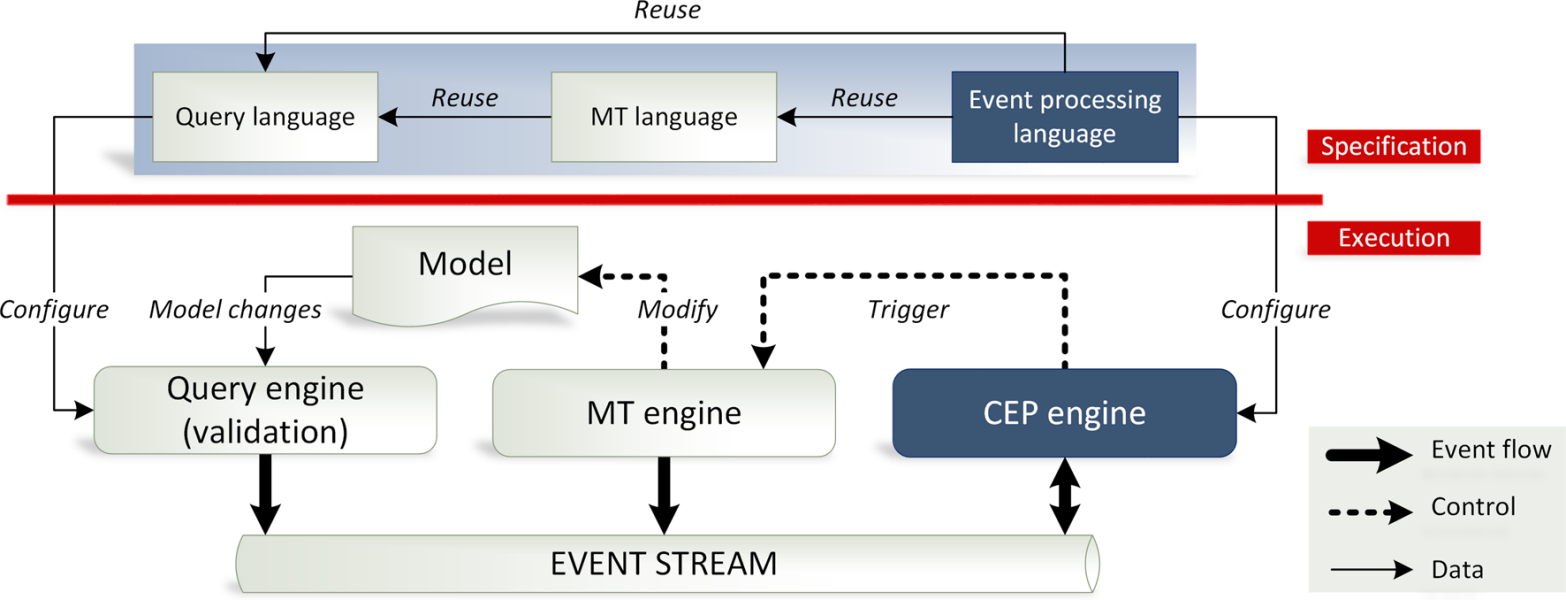
Conceptual overview of the approach with our key contributions highlighted

Standard CEP techniques do not restrict what kind of information is present in the event stream, but populating the event stream can be problematic in case of live models since atomic events can only carry information about elementary model changes, but about not compound (aggregate) changes.

The automated handling of compound model changes as atomic events of the event stream would result in (i) more simple event pattern specification and (ii) less event instances on the event stream to be processed by the CEP engine.

### Complex Patterns of Compound Changes

The main contribution of our work is an approach that allows simultaneous reasoning over *complex event sequences*, and *graphs* by incorporating both *elementary* and *compound* structural changes as events. Incremental model queries are reused to identify relevant compound structural changes and derive atomic events for an event stream which is then processed by CEP techniques. Finally, reactions observed complex events can be specified by reactive model transformation techniques. As summarized in Table 1, our approach gains advantage from (i) CDT techniques efficiently abstracting higher-level model changes into events and (ii) CEP techniques that efficiently identify sequences and temporal relations among change events.

Despite the variety of available CEP platforms and approaches, none of them supports such a deep integration with state-of-the-art model management and graph reasoning techniques (see Sect. 8 for detailed comparison). In a preliminary study [26], significant programming and integration overhead was required (both for specification and execution) to use an external CEP platform (Esper) for processing events in the context of a model transformation engine. To overcome integration problems, we developed a prototype tool with unified execution semantics of event processing and model transformations that became part of the Viatra 2 open-source Eclipse project, which offers an event-driven and reactive model transformation platform.

### Architecture

Figure 2 presents the conceptual architecture of the framework. The *Model* is continuously queried by an incremental *Query engine* with queries defined using a *Query language*. Incremental query evaluation enables to efficiently obtain the match sets of a query and to continuously track changes of the model. The match set of a query contains the set of model element tuples that satisfy the query condition.

These data are wrapped into atomic *change events* and published on an *Event stream* accessible for each component in our architecture. The *Event stream* is continuously processed by the *CEP engine* by evaluating the complex event patterns based on the processed atomic events.

Then, the *Model transformation (MT) engine* triggers reactions upon successfully matched event patterns, which includes direct manipulation of the model or publishing events to the stream. While the *Query engine* and the *MT engine* typically produces events on the stream, while the *CEP engine* both consumes and produces events

Complex event patterns are defined by the *Event processing language*, which enables to generate Java classes to represent (i) complex and atomic event patterns and (ii) atomic event classes. The latter artifacts are instantiated by event producers and define the finite language of automatically generated event types. The *Event processing language* reuses the queries defined using the *Query language* to enable referring directly to (compound) model change events; and (ii) reactive transformations defined using the *Model transformation (MT) language*.

### Architecture-Level Challenges

Although we support our approach with a dedicated tooling (presented in Sect. 6 while elaborating on the case study), one can possibly implement the architecture of Fig. 2 by making alternative technological choices for specific components, e.g., using other query, model transformation or CEP languages and engines.

In the following, we discuss the main challenges that need to be addressed to efficiently support the architecture presented in this paper.

*Graph reasoning.* A key idea in our approach is to uniformly map both elementary and compound changes of the underlying model into atomic events. Our paper uses an efficient incremental graph pattern matcher for that purpose, but in certain cases, similar results can be achieved by using a CEP engine only, where (1) atomic events carry information about elementary model changes only and (2) compound model changes are identified by the CEP engine by formulating them as complex event patterns instead of graph patterns. However, graph patterns offer a more expressive formalism for capturing structural conditions for model changes.

*Coordination of modeling languages.* Our streaming transformation approach requires at least two languages: one for complex event processing and one for model transformations. (In addition, our prototype tooling also uses a graph query language.) This clear separation of concerns raises the need for proper coordination of employed languages in order to

- allow complex event patterns and model transformations to reference each other;
- allow parameterized execution of model transformations based on matched complex event patterns;
- ensure the type safety of user-defined streaming transformation rules.

This necessitates an advanced and integrated modeling environment with rich editor support and automatic source code generation from high-level models.

*Execution challenges.* For efficient execution, the following challenges need to be addressed:

- propagation of model changes to the CEP engine;
- rule-based execution semantics for triggering model transformations based on matched complex event patterns;
- all of this in a potentially distributed way.

While our prototype uses the Viatra Event-driven virtual machine [13] as the common execution platform, where semantics is defined by traces of the underlying automata, the above tasks can be addressed by other implementations as well.

## Case study

Our motivating scenario is a synchronization problem over live models [15], which is a pertinent example of graph reasoning over non-materialized models. The synchronization process is augmented with live validation and design space exploration-based quick fix generation for invalid model states. The example is motivated by [42] and [50].

The source domain model describes a generic infrastructure for cyber-physical systems (CPS) where applications (services) are dynamically allocated to connected hosts. The target domain model represents the system deployment configuration with stateful applications deployed on hosts. We aim to derive a deployment model from the CPS model, and then, incremental model transformations are used to propagate changes in the CPS model to the deployment model.

As the source model undergoes changes (introduced by the user, for example), the CPS model might become invalid. For example, an invalid state can be reached if a model element in the CPS model is created, but its mandatory attributes are not set yet. In such cases, the automated synchronization between the CPS and the deployment model cannot proceed and manual guidance is required.

To identify invalid model states, well-formedness constraints are evaluated *continuously* over the source model, i.e., on every change. Events notifying a change in the validity (i.e., when a valid model becomes invalid or the other way round) are published on an event stream, constituting therefore an *infinite streaming model* (representing the prevailing validation state of the underlying source model).

**Fig. 3:**
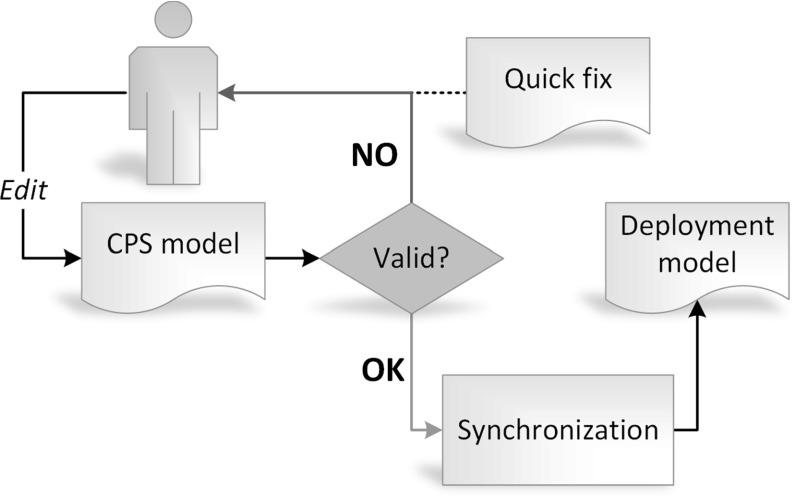
Conceptual overview of the case study

Should an invalid state be identified, quick fix suggestions are generated using design space exploration (DSE) techniques. Subsequently, the quick fixes are provided to the user in the form of a model transformation sequence to aid the process of recovering from the invalid state. The concept is illustrated in Fig. 3.

**Fig. 4:**
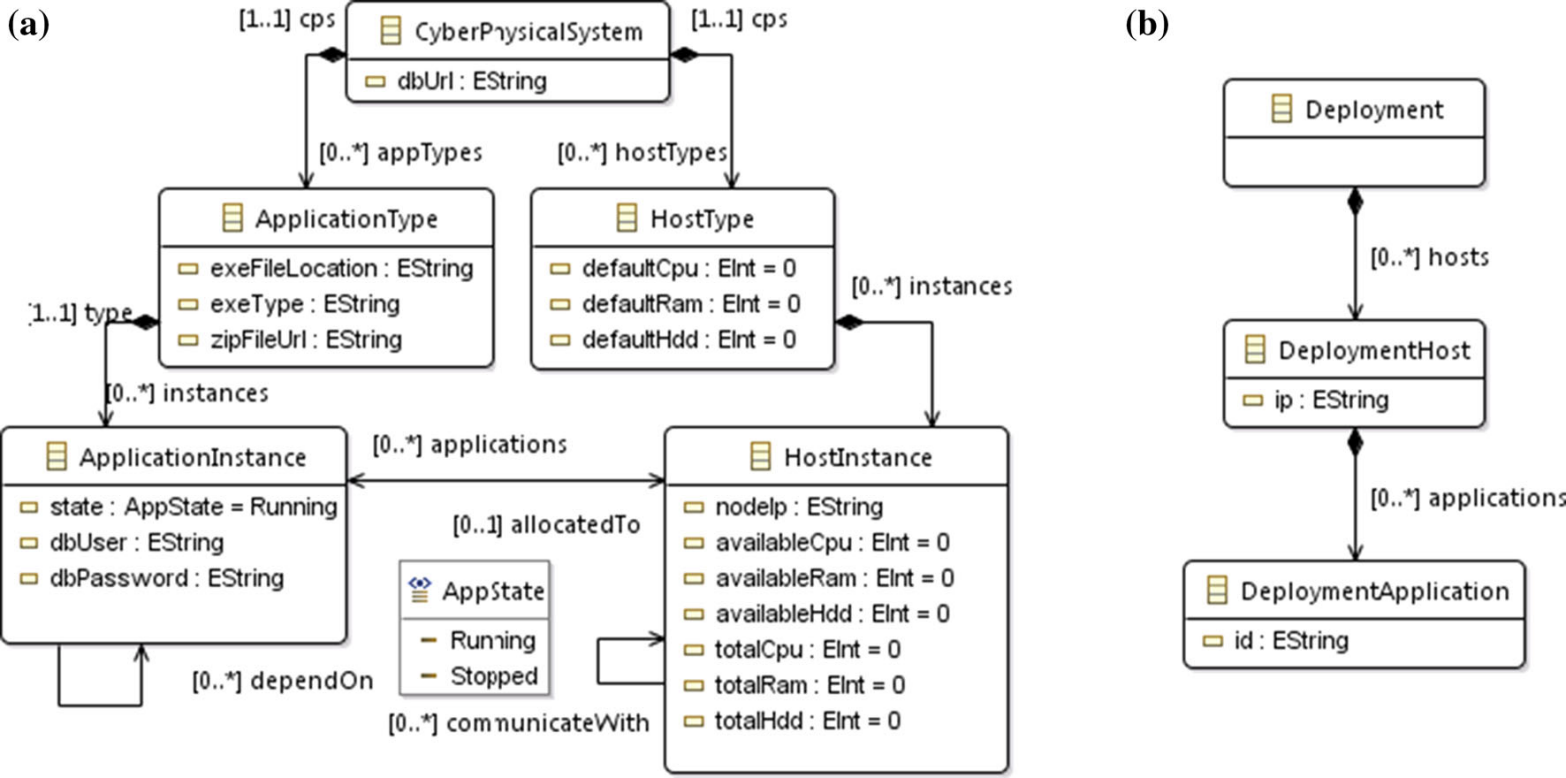
Source and target domain models. **a** Hosts and applications of the CPS. **b** Deployed hosts and applications

Meaningful units of change in the source model can be achieved typically by *non-atomic* changes. For example, adding a new model element and subsequently setting a required reference to another model element. Such a compound change can lead to an eventual validity, although during the intermediate atomic steps the model can be in an invalid state. As an advanced scenario, we aim to introduce *inconsistency tolerance* to the process, i.e., define rules over the streaming validation model which will trigger quick fix generation. Tolerance rules are depicted as *complex event patterns* and quick fixes are generated only on matches of these specific patterns. Such a pattern can be, for example, “the model being in an invalid state during five consecutive atomic changes.” Tolerance rules can be extracted from design processes, or defined manually, in both cases using suitable algebraic foundations. This problem is, however, not addressed in this paper.

*Scenarios* In the case study, the following use cases have to be addressed:

- capturing validation rules;
- modeling and processing complex patterns model validation events;
- defining quick fix generation rules in terms of design space exploration;
- integration of the components.

*Domain metamodels* Due to space considerations, we present a limited fragment of the metamodel^3^. The description of the domain (Fig. 4) is adopted from [1].

The simplified CPS source model (Fig. 4a) contains *HostInstance*s and *ApplicationInstance*s, typed by *HostType*s and *ApplicationType*s, respectively. *ApplicationInstance*s are allocated to a *HostInstance*. In the Deployment model (Fig. 4b), *DeploymentHost*s and *DeploymentApplication*s are derived from their CPS model counterparts, respectively, and the hosts are associated with the hosted applications. *HostInstance*s provide CPU, RAM and HDD capabilities to the CPS. These parameters are characterized by an *available* and a *total* amount. A typical validation rule would check whether the available amount of a given resource type is lower than the total.

## Language structure and semantics

This chapter summarizes the syntax and the formal semantics of our event processing language. The language is highly motivated by the currently available ones in the CEP domain (in particular [21]), but with more focus on change events of engineering models.

### Syntax

The language is built up from a finite set of

- atomic event patterns $\mathcal {A}$ referring to elementary events (observed on an event stream), and
- complex event patterns $\mathcal {C}$ defining complex event sequences constructed by
- complex event operators $\mathcal {O}$: $\mathtt {fol}$, $\mathtt {or}$, $\mathtt {and}$, $\mathtt {mult}$, $\mathtt {\lnot }$ (negative application condition—NAC), $\mathtt {win}$.

#### Definition 1

Every **atomic event pattern** $a \in \mathcal {A}$ is a pair $(t, {\varPhi })$ where *t* is an event type and ${\varPhi }$ is a list of formal parameters.

A **query event pattern** (QEP) is a special subtype of atomic event patterns, which represents a change events of continuously evaluated query results over a model. A query event pattern, therefore, extends the definition of the atomic event pattern: $a_q \in \mathcal {A}_q \subseteq \mathcal {A}$ is a 4-tuple $(t, {\varPhi }, t_q, t_{ch})$, where $t_q$ is the unique type (name) of the query and $t_{ch}$ is the type of the change, with $t_{ch} \in \{Appear, Disappear\}$. $\square$

#### Definition 2

A **complex event pattern** $c \in \mathcal {C}$ is defined as $(t,{\varPhi }, Body)$ where *t* is a (unique) type of the event, ${\varPhi }$ is a list of formal parameters and *Body* is inductively defined as follows:

- $\underline{Body := a}$. Atomic event pattern $a=(t, {\varPhi })$ implies a complex event pattern $c=(t,{\varPhi }, \emptyset )$ with corresponding parameters; or
- $\underline{Body := op(c_1,c_2).}$ $c_1$ and $c_2$ are complex event patterns then $op(c_1,c_2)$ is a complex event pattern where *op* is a complex event operator from the set $\mathcal {O} = \{ \mathtt {fol},\, \mathtt {or},\, \mathtt {and},\, \mathtt {mult},\, \mathtt {win},\, \mathtt {\lnot } \}$. $\square$

The latter definition can easily be extended to allow operations for a *sequence* of complex event types (instead of binary complex event operators), but we restrict the notations of the paper to binary operators to simplify presentation and handle this as a syntactic sugar of our language.

### Atomic Event Instances in Event Streams

#### Definition 3

An **atomic event instance** $e \in \mathcal {E}_\sigma$ is an observable entity on some event stream $\sigma \in {\varSigma }$. Atomic event instances are defined as $e = (t, {\varPsi }, \tau )$, i.e., by their *type*, *list of parameter values* (${\varPsi }$) and *timestamp* of appearance ($\tau$), respectively. We denote the different components of an atomic event instance as *e*.*t*, $e.{\varPsi }$ and $e.\tau$, respectively. $\square$

In the scope of the current paper, we do not distinguish between different event streams and process events aggregated from all of the event streams instead. Thus, the language of all observable atomic event instances is: $\mathcal {E} = \bigcup \limits _{\varSigma } \mathcal {E}_\sigma$.

#### Definition 4

$E_1^n$ denotes the **sequence of observed atomic event instances**. That is, $E_1^n = e_1, e_2 \ldots e_n$, where $\forall i \in \mathbb {N} : e_i \in \mathcal {E}$. $\square$

#### Definition 5

An atomic event pattern is **matched over an event stream** iff an atomic event instance with the appropriate type is observed on the event stream. Formally, $E_1^n \models a \in \mathcal {A}$ iff $\exists e \in E_1^n: e.t = a.t$. We also use the shorthand notation $e \models a \in \mathcal {A}$ iff $e \in E_1^n ~\wedge ~ E_1^n \models a ~\wedge ~ e.t = a.t$. $\square$

Atomic event patterns can only feature *output* parameters, where the parameters of an atomic event pattern match are bound from the observed atomic event instance. Formally, $\forall e \in E_1^n, a \in \mathcal {A}, e \models a: a.{\varPhi } \leftarrow e.{\varPsi }$.

*Query event patterns.* The atomic event instances required to match a query event pattern originate from a model query engine. Characteristic changes in the life cycle of a model query match (such as appearance, update, disappearance) are labeled and atomic event instances are generated upon these phases.

#### Definition 6

A query event pattern is matched iff an atomic event instance with the appropriate type is observed on the event stream, and the life cycle change of the referred model query match is in line with the one defined in the pattern. Formally, $E_1^n \models a_q \in \mathcal {A}_q$ iff $\exists e \in E_1^n: e.t = a_q.t ~\wedge ~ e.t_{ch} = a_q.t_{ch}$. $\square$

### Semantics of Complex Event Patterns

As opposed to atomic event instances, complex event instances cannot be directly observed on the event stream. Instead, the latter types of events are modeled and inferred from the stream of atomic event instances using an appropriate event algebra.

#### Definition 7

**Match of a complex event pattern** In general, a complex event pattern is (fully) **matched** if and only if (i) each of its referred sub-patterns are matched and (ii) parameter bindings of atomic sub-patterns can be successfully unified [10]. Formally,

- $$E_1^n \models c \in \mathcal {C}$$ iff $$\forall c' \subset c: \exists E_i^j \subseteq E_1^n \models c'$$, and
- $$\forall \phi _1, \phi _2 \in \bigcap \limits _{c' \subset c}{\varPhi }_{c'}: \phi _1 \equiv \phi _2$$. $$\square $$

The precise and executable semantics of matching a complex event pattern over an event stream is defined by

- the semantics of the complex event operator (*op*), used in the pattern (Sect. 4.3.1); and
- the event processing context (Sect. 4.4).

#### Definition 8

**Partial event pattern match** An event pattern is partially matched if at least one of its sub-patterns is matched, but at least one of its sub-patterns is not matched. This relation is denoted by $\models _p$.

Formally,

$$E_1^n \models _p c \in \mathcal {C}$$ iff $$\exists c_1, c_2 \subset c:$$

- $$\exists E_i^j \subseteq E_1^n \models c_1$$, but
- $$\not \exists E_i^j \subseteq E_1^n \models c_2$$. $$\square $$

#### Definition 9

The **timestamp of a complex event pattern match** is the timestamp of the last sub-pattern being matched. Formally, if $E_1^{n-1} \nvDash c$, but $(E_1^{n-1}; e_n) \models c$, then $c.\tau :=e_n.\tau$.

Here, $(E_1^{n-1};e_n)$ denotes the event $e_n$ being appended to the end of the sequence $E_1^{n-1}$ and $\forall e_i \in E_1^{n-1}: e_i.\tau \le e_n.\tau$ holds. $\square$

#### Operator Semantics

Based on the definitions of the static structure (Sect. 4.1), we define the operators of our event processing algebra.

- **Followed by**: $\mathtt {fol}(c_1, c_2)$ $E_1^n \models \mathtt {fol}(c_1, c_2)$ iff $E_1^n \models c_1 ~\wedge ~ E_1^n \models c_2$, where $c_1.\tau <c_2.\tau$, i.e., the pattern is matched if and only if every sub-pattern is matched, and in the specific order defined by the pattern.
- **Or**: $\mathtt {or}(c_1, c_2)$ $E_1^n \models \mathtt {or}(c_1, c_2)$ iff $E_1^n \models c_1 \vee E_1^n \models c_2$, i.e., the pattern is matched if and only if one of the sub-patterns is matched.
- **And**: $\mathtt {and}(c_1, c_2)$ $E_1^n \models \mathtt {and}(c_1, c_2)$ iff $E_1^n \models c_1 ~\wedge ~ E_1^n \models c_2$, i.e., the pattern is matched if and only if every sub-pattern is matched. The $\mathtt {and()}$ operator is a syntactic sugar, formally defined as the combination of the $\mathtt {fol()}$ and $\mathtt {or()}$ operators: $\mathtt {and}(c_1, c_2) \equiv \mathtt {or}(\mathtt {fol}(c_1, c_2), \mathtt {fol}(c_2, c_1))$.
- **Multiplicity**: $\mathtt {mult}(c, n)$ $\forall c \in \mathcal {C}, n \in \mathbb {Z}^+: E_1^n \models \mathtt {mult}(c, n)$ iff $E_1^n \models \mathtt {fol}(c_1^n)$. That is, the pattern is matched if and only if *n* occurrences of pattern *c* are matched. Specifically,
  - the *Arbitrary* operator: $E_1^n \models \mathtt {mult}(c, *)$ iff $E_1^n \models \mathtt {mult}(c, n), n\ge 0$;
  - the *At least once* multiplicity operator: $E_1^n \models \mathtt {mult}(c, +)$ iff $E_1^n \models \mathtt {mult}(c, n) ~\wedge ~ n\ge 1$.
  Note that the former operator also allows *no occurrence* of *c*; hence, this operator cannot be applied on atomic events, since it would match *empty* patterns. Additionally: $\mathtt {mult}(c, +) \equiv \mathtt {fol}(c, \mathtt {mult}(c, *))$.
- **Time window**: $\mathtt {win}(\mathtt {fol}(c_1, c_2), {\varDelta }, ws)$ Applying a time window $\mathtt {win}$ of time window semantics *ws* and of length ${\varDelta }$ on the complex event pattern *c* intuitively means the following. Let $c_1$ denote the leftmost and $c_2$ denote the rightmost sub-pattern of the pattern. Using this notation, the following rules apply:
  - $$E_1^n \models \mathtt {win}(\mathtt {fol}(c_1, c_2), {\varDelta }, Within)$$ iff $$E_1^n \models \mathtt {fol}(c_1, c_2) ~\wedge ~ |c_1.\tau - c_2.\tau | \le {\varDelta }$$.
  - $$E_1^n \models \mathtt {win}(\mathtt {fol}(c_1, c_2), {\varDelta }, HoldsFor)$$ iff $$E_1^n \models \mathtt {fol}(c_1, c_2) ~\wedge ~ |c_1.\tau - c_2.\tau | \ge {\varDelta }$$.
  The time  window operator is only applicable to $\mathtt {fol}$ constructions, or to those available to be expressed via such a construct. In the current set of operators, this means the $\mathtt {and}$ and the $\mathtt {mult}$ operators. To efficiently handle time window constraints of arbitrarily complex event patterns, we investigate the algebraic axioms of the operators and we conclude a general rule to this end.
- **Negative application condition (NAC)**: $\lnot {c}$ $\forall c \in \mathcal {C}: E_1^n \models \lnot {c}$ iff $E_1^n \nvDash c$. The distributive nature of the NAC operator over the $\mathtt {fol}(c_1, c_2)$ and $\mathtt {or}(c_1, c_2)$ operators:
  - $$\lnot (\mathtt {fol}(c_1, c_2)) \equiv \mathtt {or}(\lnot {c_1}, \mathtt {fol}(c_1, \lnot {c_1}))$$
  - $$\lnot (\mathtt {or}(c_1, c_2)) \equiv \lnot (c_1) \wedge \lnot (c_2)$$
  Applying the NAC operator on multiplicities: $\forall c \in \mathcal {C}, n \in \mathbb {Z}^+: E_1^n \models \lnot (\mathtt {mult}(c, n))$ iff $E_1^n \models \mathtt {fol}(c_1^m) \wedge m<n$. That is, the pattern is matched if and only if *n* occurrences of pattern *c* are *not* matched, i.e., the pattern is matched a maximum of *m = n-1* times. Consequently,
  - $\lnot (\mathtt {mult}(c, *))$ is not defined, because of $m < 0$;
  - $$\lnot (\mathtt {mult}(c, +)) \equiv \lnot {c}$$, because of $$m < 1$$.
  Applying the NAC operator to a time window operator switches the time window semantics from *Within* to *HoldsFor*, and the other way around.

#### Interaction of Operators

Operator precedence rules divide operators into two groups which the precedence rules are defined between. However, there are no precedence rules defined within the groups.

- Higher precedence operators are: $\mathtt {mult}$, NAC, $\mathtt {win}$.
- Lower precedence operators are: $\mathtt {fol}$, $\mathtt {or}$, $\mathtt {and}$.

In general, the group of operators with lower precedence are the binary operators of the algebra, while the operators of higher precedence are the unary ones.

Algebraic axioms [31] define logical transformation rules between differing algebraic structures. These transformations are useful when the validity of complex event patterns needs to be assessed. Table 2 summarizes the characteristic properties of the binary operators of our event algebra. The propositions and proofs are available in “Appendix 1.”

**Table 2:** Algebraic axioms of the complex event operators (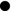/ 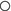: relationship does/does not hold)

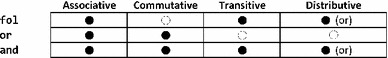	

*Axioms for the time window operator.* Although all the binary operators are associative (in both directions) by nature, to efficiently handle time window constraints, we introduce the following convention.

##### Definition 10

The evaluation of the $\mathtt {or}$ and $\mathtt {and}$ complex event operators follows a **left-associative** convention, while the evaluation of the $\mathtt {fol}$ operator follows a **right-associative** convention. That is, the following *rewriting rules* (denoted by $\leadsto$) apply,

- $$\mathtt {or}(c_1, c_2, c_3) \leadsto \mathtt {or}(\mathtt {or}(c_1, c_2), c_3)$$;
- $$\mathtt {and}(c_1, c_2, c_3) \leadsto \mathtt {and}(\mathtt {and}(c_1, c_2), c_3)$$; but
- $$\mathtt {fol}(c_1, c_2, c_3) \leadsto \mathtt {fol}(c_1, \mathtt {fol}(c_2, c_3))$$. $$\square $$

Making the $\mathtt {fol}$ operator right-associative is motivated by the following proposition.

##### Proposition 1

Assuming right-associativity, $\mathtt {win}(\mathtt {fol}(c_1, c_2, c_3), {\varDelta }, ws)$ can be rewritten into: $\mathtt {fol}(c_1, c_2, c_3) ~\wedge ~ \mathtt {win}(\mathtt {fol}(c_1, c_3),{\varDelta }, ws)$. $\square$

This concludes that evaluating time window constraints requires only comparing the timestamps of the rightmost and leftmost sub-patterns.

##### Proof

Following Definition 9, as the patterns are evaluated from right to left, it is always the rightmost sub-pattern that determines the timestamp of the complex event pattern. $\square$

### Event Processing Contexts

Observed event instances might contribute to multiple complex event pattern instances. Specifying which partial event pattern match(es) an observed atomic event instance is allowed to contribute to, is achieved by using *event processing contexts* [21], or event contexts, in short. Cugola et al. [24] refer to the concept as *consumption rules*.

The event context is a global parameter to the specific event processing task.

**Fig. 5:**
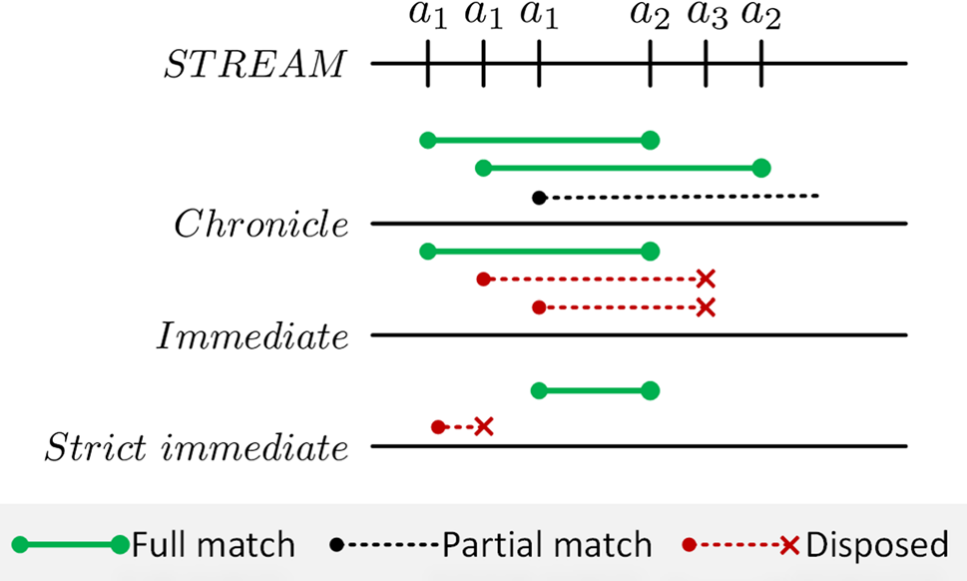
Matches of the $\mathtt {fol}(a_1, a_2)$ pattern under different event contexts, given a sequence of observed event instances $a_1, a_1, a_1, a_2, a_3, a_2$

Figure 5 shows the three-event processing contexts discussed in this paper. The figure shows how event processing contexts influence the evaluation of the $\mathtt {fol}(a_1, a_2)$ pattern over an example stream of events, consisting of the following event instances: $a_1, a_1, a_1, a_2, a_3, a_2$. As the figure shows, different event contexts result in differently matched event pattern instances.

To formalize event processing contexts, we use the concept of partial event match sets.

#### Definition 11

**Set of partial event pattern matches** Let *P* denote the set of partial event pattern matches of any defined complex event pattern $c \in \mathcal {C}$; and $P_c \subseteq P$ denote the set of partial event pattern matches of a complex event pattern $c \in \mathcal {C}$, at a given point of time. (Consequently, $P = \bigcup \limits _{c \in \mathcal {C}} P_c$.)

Additionally, let $P(e) \subseteq P$ and $P_c(e) \subseteq P_c$ denote the set of partial event pattern matches that the observed event *e* can contribute to. $\square$

*Chronicle* We use the Chronicle context in accordance with [21]. This context enables tracking arbitrary number of event patterns and uses every atomic event instance in exactly one event pattern. Event instances are considered in the order they appeared, i.e., the observed event instance *e* is always associated with the oldest partial event pattern instance. Formally, $chronicle(P, e): e \mapsto \underset{\tau _{start}}{\min }(P)$.

As Fig. 5 shows, two event pattern instances are matched in the example using this context: $a_1(1)-a_2(1)$ and $a_1(2)-a_2(2)$, while a partial event pattern instance is still unmatched.

*Immediate* In some scenarios, e.g., in our gesture recognition case study in Sect. 7, noise on the event stream(s) is required to be taken into account. By noise with respect to a complex event pattern, we generally mean an observed event instance not contributing to the specific complex event pattern. Formally, event *e* is considered as noise with respect to complex event pattern $c \in \mathcal {C}$ iff $P_c(e) \equiv \emptyset$.

The Immediate context extends the definition of the Chronicle context by defining how to deal with noise. In case of a noise event, every partial event pattern is disposed by definition. Formally, $immediate(P, e): P_c(e) \equiv \emptyset \Rightarrow P:= \emptyset$.

In the example in Fig. 5, this results in two partial event pattern instances being disposed upon observing $a_3$, as it does not contribute to the pattern itself. The example also explains the naming, as partially matched event patterns are required to evolve *immediately* after an event is observed on the event stream.

*Strict immediate* The Strict immediate context restricts the Immediate context by allowing only one match to be tracked at the same time per complex event pattern. Formally, $strict(c): \left| {P_c}\right| \le 1$.

This restriction leads to more aggressive noise filtering. In the example, the first instance of $a_1$ starts a partial complex event instance, and since this is the only one allowed to be tracked in this context, the second instance of $a_1$ cannot contribute to any pattern instance, hence it is considered as noise. Finally, the $a_1(3)-a_2(1)$ pattern instance will match.

## Executable Semantics

**Fig. 6:**
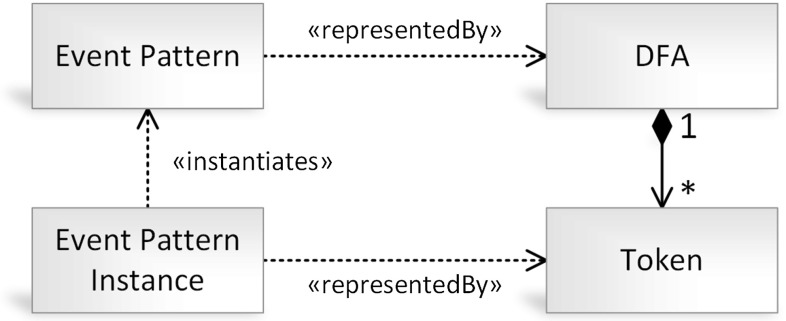
Mapping between event patterns and automata

To enable the execution of the Vepl language, the event processing algebra, its operators and logical structures are required to be mapped to an appropriate formal representation. In the case of keeping track of partially and fully matched phases of single event patterns, automaton-based formalisms seem to be a natural fit. We chose a *deterministic finite automaton* (DFA) [47]-based representation in which states represent the phases of pattern matching, while tokens represent specific event pattern matches passing through the different phases. This concept is highlighted in Fig. 6.

In this section, we discuss the underlying DFA structure and its extensions to support evaluating time windows and formal parameters of event patterns.

### Structure of the Underlying DFA Formalism

An automaton *M* is a 7-tuple ($Q, q_0, f, x, \mathcal {E}, \delta , T$), consisting of

- a finite set of states (*Q*);
- an initial (start) state ($q_0 \in Q$);
- a final (accept) state ($f \in Q$);
- a trap state ($$x \in Q$$);
- a finite set of input event types (the alphabet) ($\mathcal {E}$);
- a transition function ($$\delta : Q \times \mathcal {E} \mapsto Q$$);
- a set of timed zones (*T*).

*States.* The **states** (*Q*) represent the relevant phases of detecting the complex event pattern and *tokens* represent the (partial or complete) matches of complex event patterns.

#### Definition 12

A state $q \in Q$ is said to be an **intermediate state** of an automaton if the state is neither an initial nor final nor a trap state. Formally, $q \not \in \{q_0, f, x\}$. $\square$

The completeness of an event pattern match is determined by the state its token is placed at. A token in the *initial state* $q_0$ represents the state where no event contributing to the complex event pattern has been observed yet, while a token in the *final state* *f* represents a full event pattern match. The *trap state* *x* is a special state indicating that a complex event pattern can never be matched, e.g., the expiration of a time window could result in such an error. *Intermediate* states represent partial phases of an event pattern match. 
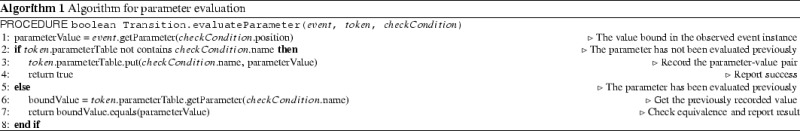


*Language of input events.* The **language of input events** $\mathcal {E}$ follows the definition provided in Sect. 4.2.

*Transition function.* The **transition function** $\delta$ defines how the pattern matching process can evolve from one phase to another, i.e., proceed over states in *Q*. The transition function is determined by the operator and the type of the referred event types in the complex event pattern. The former one determines in what structure transitions interweave states of the automaton, while the latter information is used to define *guards* for transitions. In general, every transition is typed by exactly one atomic event type and is enabled if an instance of that atomic event type is observed on the stream.

Transitions are also responsible for triggering the unification-style evaluation of the formal parameters of event patterns, as presented in Definition 7. At compilation time, transitions are augmented with information to evaluate parameter bindings, which is a map of *string*-*integer* pairs, each pair referring to

- the symbolic name of the parameter in the complex event pattern and
- the position of that symbolic parameter in the given atomic sub-pattern, respectively.

The parameter evaluation behaves like an additional guard in addition to the atomic event type the transition is typed with. Once the type is successfully matched and a token attempts to transition, the parameters are evaluated, as shown in Algorithm 1.

1. First, the value bound to the parameter in the current event instance is obtained (Line 1).
2. If the parameter table of the token does not contain previously bound values to the given symbolic name (Line 2), the equality criteria will not be violated. Therefore, the value is bound to the symbolic name for the first time and it is persisted in the parameter table of the token (Line 3).
3. If the parameter table contains a record with the given symbolic name (Line 5), its previously bound value is obtained (Line 6), compared to the value bound in the observed event instance and the result is reported (Line 7).

*Negative transitions* typed by an event pattern, represent NAC expressions and are evaluated by the rule defined in Section 4.3.1. That is, $\forall E_1^n \models \lnot {a}$ iff $E_1^n \nvDash a$, i.e., the negative transition guarded by an atomic event type is fired iff the last-observed atomic event instance has a mismatching type.

*Timed zones.* The **timed zones** (*T*) represent time window constraints ($\mathtt {win}$) on the level of the automaton. Formally, a timed zone is defined as a 4-tuple ($Q^t, Q_{in}^t, Q_{out}^t, tw$), consisting of

- the states within the timed zone $Q^t$;
- the in-states of the timed zone $Q_{in}^t$;
- the out-states of the timed zone $Q_{out}^t$;
- the length of the timed zone *tw*.

**Fig. 7:**
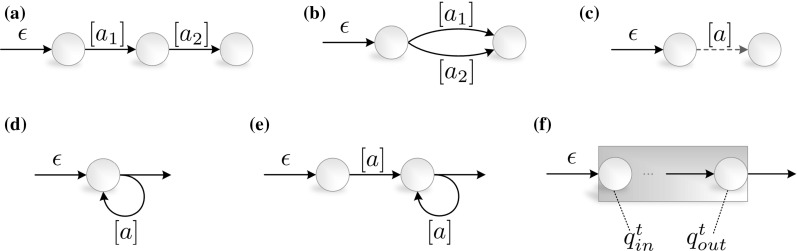
Patterns of mapping complex event operators to DFA. (a) $\mathtt {fol}(a_1, a_2)$. (b) $\mathtt {or}(a_1, a_2)$. (c) $\mathtt {not}(a)$. (d) $\mathtt {mult}(a, *)$. (e) $\mathtt {mult}(a, +)$. (f) $\mathtt {win}(c, tw)$

States of the automaton can be associated with multiple timed zones. As tokens are passed across a timed zone, first they enter the zone by marking one of the in-states ($Q_{in}^t$) and then leave the zone by marking one of the out-states ($Q_{out}^t$). Timestamps of both these events are recorded by the token. Upon attempting to leave a timed zone, the two timestamps are compared to *tw* as defined in Sect. 4.3.1. If the appropriate time window condition holds, the token can leave the timed zone; otherwise, it is placed into the trap state ($x \in Q$).

Figure 7 shows the patterns of mapping the operators of the event algebra on automaton structures. It can be concluded that every pattern generates a structure being both deterministic and finite; therefore, every automaton will be a DFA indeed.

*Steps and traces of an automaton.* The execution of an automaton is defined as a sequences of steps called traces.

#### Definition 13

**Marking of an automaton** The $\mu : Q(M) \mapsto Z$ state multiset captures a configuration of automaton *M*, where *Z* denotes the set of tokens *z* assigned to the set of set states *Q*. $\mu (q)$ denotes the current marking of state $q \in Q$. $Z_{\mu }(q)$ denotes the set of tokens assigned to state $q \in Q$ in the configuration defined by $\mu$. $\square$

#### Definition 14

**Step of an automaton** A step of an automaton *M* is defined as $\xi : (\mu _0, e \in \mathcal {E}) \mapsto \mu _1$, where

- $$\exists q_1, q_2 \in Q, q_1 \ne q_2, z \in Z: z \in Z_{\mu _0}(q_1) ~\wedge ~ z \in Z_{\mu _1}(q_2)$$, and
- $$\forall q \in Q, z' \in Z\backslash z: z' \in Z_{\mu _0}(q) \Rightarrow z' \in Z_{\mu _1}(q)$$.

That is, a step captures a change in the marking of the automaton triggered by an observed atomic event instance; the change involves exactly one token being assigned to a new state, but all the other tokens being left intact. $\square$

#### Definition 15

**Trace of an automaton** By a trace *t* of an automaton *M*, we mean the ordered sequence of steps: $t = \{\xi _1, \xi _2 \ldots \xi _n\}$. We use the notation $(M, E_1^n) \underset{t}{\vdash }q \in Q$ to express that given an automaton *M* and an input event stream $E_1^n$, $\mu (q)$ can be inferred through a trace *t*, and the *t* begins with a step $\xi _1$ moving a token from the initial state. $\square$

### Completeness and Soundness of the Mapping

Below we show that the proposed DFA-based execution model, i.e., the mapping from the structures of the Vepl language to DFAs is complete and sound with respect to its semantics defined in Sect. 4.

#### Definition 16

**Completeness and soundness of a mapping** Let $M=map(c \in C)$ be an automaton identifying the complex event *c* and $f \in Q$ denote the final state of the automaton. Then

- a mapping $M = map(c \in C)$ is complete if: $E_1^n \models c \Rightarrow \exists f \in Q, t: (M, E_1^n) \underset{t}{\vdash }f$; and
- a mapping $M = map(c \in C)$ is sound if: $\exists f \in Q, t: (M, E_1^n) \underset{t}{\vdash }f \Rightarrow E_1^n \models c$. $\square$

#### Proposition 2

The mapping $M = map(c \in C)$ is always complete. $\square$

#### Proof

Let $E_i^j \subseteq E_1^n = e_i$ ...$e_j$ be the timestamp-ordered sequence of atomic event instances constituting the complex event pattern match. That is, $\{e_i$ ...$e_j\} \models c$.

Due to the construction algorithm of the automaton *M*, $\forall e_i ~\exists q_1, q_2 \in Q: \delta (q_1, e_i) \mapsto q_2$, where for the state pair $(q_1, q_2) \in t$ holds always. Specifically, in the sub-cases of complex event operators defined in Sect. 4.3.1 and the mapping patterns in Fig. 7:

- $$\mathtt {fol}(e_{i-1}, e_i)$$: $$\exists q_0, q_1, q_2 \in Q:$$
  - $$\delta (q_0, e_{i-1})\mapsto q_1 ~\wedge ~$$
  - $$\delta (q_1, e_i)\mapsto q_2$$;
- $$\mathtt {or}(e_{i-1}, e_i)$$: $$\exists q_0, q_1 \in Q:$$
  - $$\delta (q_0, e_{i-1})\mapsto q_1 ~\vee ~$$
  - $$\delta (q_0, e_i)\mapsto q_1$$;
- $$\mathtt {and}(e_{i-1}, e_i)$$: $$\exists q_0, q_1, q_1', q_2 \in Q:$$
  - $$\delta (q_0, e_{i-1})\mapsto q_1 ~\wedge ~ \delta (q_1, e_i)\mapsto q_2 ~\vee ~$$
  - $$\delta (q_0, e_i)\mapsto q_1' ~\wedge ~ \delta (q_1', e_{i-1})\mapsto q_2$$;
- $$\mathtt {\lnot }(e_i)$$: $$\exists q_0, q_1, x \in Q:$$
  - $$\delta (q_0, e_i)\mapsto x ~\wedge ~ \delta (q_0, \mathcal {E} \backslash e_i)\mapsto q_1$$.

Inductively, by applying the $\delta$ transition function $j-i$ times with respect to the $E_i^j$ input stream, the trace leads from $q_0$ to *f*. Consequently, $E_1^n \models c \Rightarrow \exists f \in Q, t: (M, E_1^n) \underset{t}{\vdash }f$. $\square$

#### Proposition 3

The mapping $M = map(c \in C)$ is always sound. $\square$

#### Proof

$$\forall \{q_1, q_2\} \subseteq t: \exists \delta (q_1, e_i) \mapsto q_2$$, where $$e_i \in \mathcal {E}$$.

Depending on the structure of the sub-graph spanned by $q_1$ and $q_2$:

- $\forall e_i, e_i' \in \mathcal {E} ~\exists \delta (q_1, e_i)\mapsto q_2 ~\wedge ~ \exists \delta (q_1, e_i')\mapsto q_2 \Rightarrow e_i = e_i'$, i.e., there is only one transition between two states, then the structure models $e_1$ and hence, by appropriate concatenation it models $\mathtt {fol}(e_{i-1}, e_i)$.
- $\forall e_{i-1}, e_i \in \mathcal {E}, e_{i-1} \ne e_i ~\exists \delta (q_1, e_{i-1})\mapsto q_2 ~\wedge \exists \delta (q_1, e_i)\mapsto q_2$, i.e., there are two or more identically directed transitions between two states, then the structure models $\mathtt {or}(e_{i-1}, e_i)$.
- $\forall e_i \in \mathcal {E}, x \in Q ~\exists \delta (q_1, e_i)\mapsto x ~\wedge ~ \exists \delta (q_1, \mathcal {E} \backslash e_i)\mapsto q_2$, i.e., there is one transition with the given event type directing to the trap state and there is one transition with the negation of the given event type directing to a non-trap state, then the structure models $\mathtt {\lnot }(e_i)$.

By iterating through the elements of the *t* trace, it will imply the series of events $e_i \ldots e_j \in E_1^n \models c$. Consequently, $\exists f \in Q, t: (M, E_1^n) \underset{t}{\vdash }f \Rightarrow E_1^n \models c$. $\square$

## Elaboration of the Case Study

**Fig. 8:**
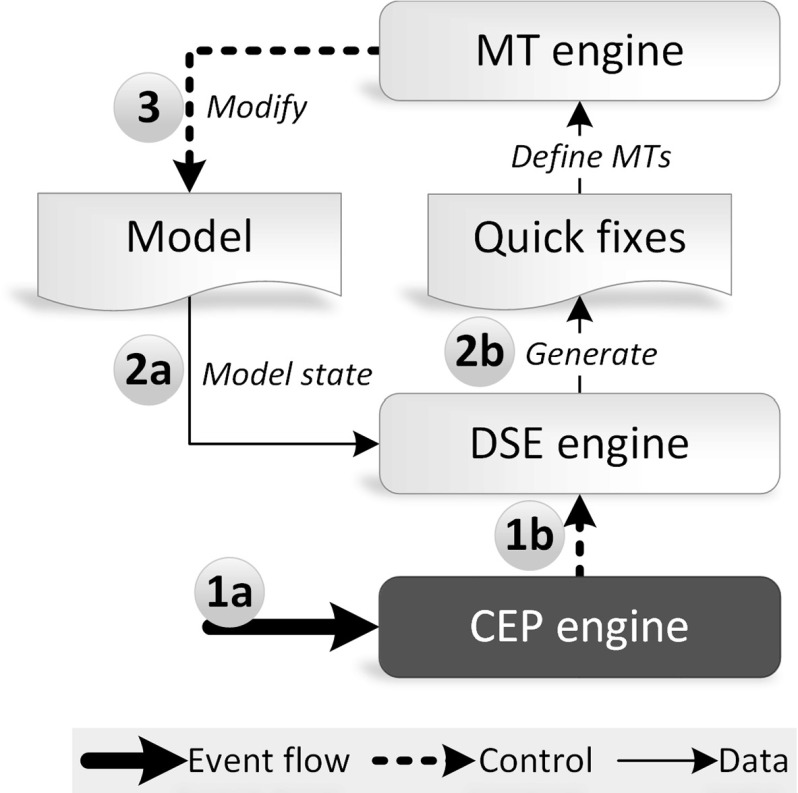
Detailed overview of the reactive workflow

In this section, we demonstrate how streaming transformations can be defined by building upon query and transformation languages by elaborating on the case study.^4^ Figure 8 shows the detailed overview of the case study in accordance with Fig. 2.

- **Phase 1a** Atomic event instances are processed by the CEP engine. These events reflect changes in the validity of the observed model, originating from a query engine.
  - First, *model queries* are defined to depict well-formedness rules of the source model. Appearance and disappearance of query matches represent changes in the validity of the model.
  - To process atomic (change) event instances, *atomic and complex event patterns* are defined using the Vepl language. Complex event patterns identify states of the model in which intervention is required, i.e., when a toleration limit of invalid model state is reached.
- **Phase 1b** When the appropriate complex event pattern is matched, the DSE engine should be notified. This is achieved by defining *actions* and associating them with appeared matches of complex event patterns.
- **Phase 2a-2b** As the DSE engine is notified, it queries the model state and generates quick fixes, defining model transformation alternatives the user can select from. The DSE engine is configured by the appropriate *objectives* and *transformation rules*.
- **Phase 3** After choosing one of the quick fixes, a model transformation is executed on the model.

The stream of atomic validation event instances constitutes the streaming model under processing. The model transformations are driven by complex event patterns inferred from this stream and are executed upon the underlying source model.

*Technological choices* To tackle the laborious and error-prone efforts of tool integration, we use the Viatra platform^5^ for graph querying, event processing, design space exploration and model transformation purposes as well.

The Viatra-CEP [27] event processing framework is designed to efficiently support advanced *modeling* scenarios. Its event processing DSL, the Viatra Event Processing Language (Vepl) implements the ideas presented in Sect. 4.

**Fig. 9:**
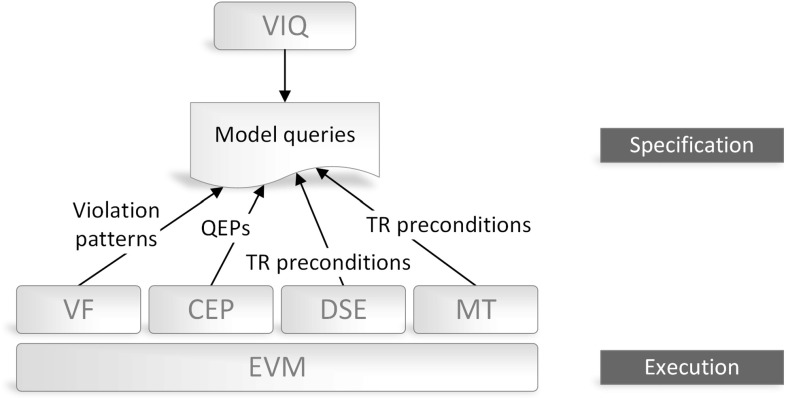
Specification- and execution-phase interplay between various components of the Viatra stack

As shown in Fig. 9, the CEP framework integrates with both the Viatra-DSE design space exploration framework [1, 43] and the Viatra-MT model transformation engine [13]. Model queries are captured using the Viatra Queries framework (formerly EMF-IncQuery) [70] in all the three subsequent steps of CEP, DSE and MT, and are evaluated in an incremental fashion. Additionally, the ViatraEvent-driven Virtual Machine (EVM) [13] serves as the unified and reactive environment governing the various phases of execution.

**Fig. 10:**
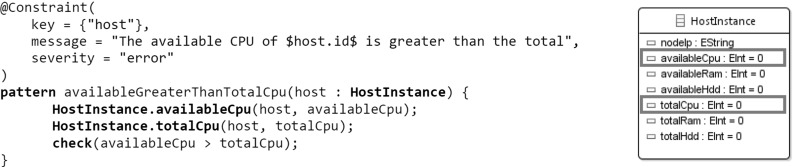
Validation rule detecting when the *availableCpu* is greater than the *totalCpu* of a *HostInstance*

**Fig. 11:**
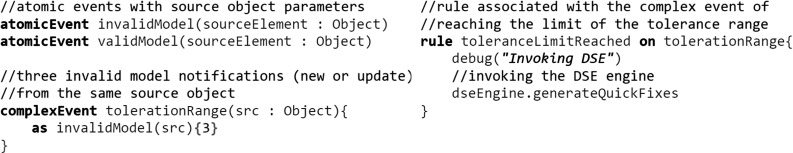
Event patterns and a rule associating the quick fix generation with the match of the complex event pattern

### Model Queries for Structural Constraints

Model queries capture structural constraints of a model. Fig. 10 shows an example of a validation rule defined by a graph pattern depicting an invalid state of the model. The pattern is parameterized with a HostInstance and checks whether its availableCpu property is greater than the totalCpu, which is clearly a validity violation, as the former property can never be greater than the latter one on the same HostInstance.

The query is augmented with information from the Validation framework [51] of the Viatra Queries framework. This information is introduced by the @Constraint annotation and is parameterized by a reference to the source model object (host), a validation message and the severity of the issue. (error, in this case.)

The framework allows defining violation listeners for single validation rules. Violation listeners are updated on every state change of the related violation rule and facilitate retrieving validation information at run-time. We use this facility to generate atomic event instances, wrap violation updates into these events and subsequently, publish them on an event stream.

**Fig. 12:**
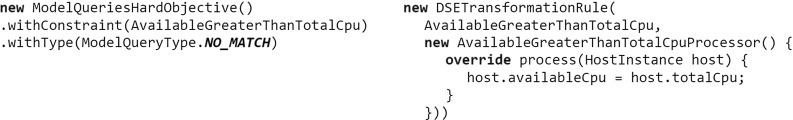
DSE objective and transformation rule reusing the previously defined Viatra Queries patterns

### Defining Atomic and Complex Events

In the next step, atomic and complex event patterns are defined. As shown in Fig. 11, two atomic event patterns are defined to depict events of the underlying model being in an invalid and in a valid state, respectively. An invalidModel event is generated if a previously valid model becomes invalid. The validModel atomic event depicts the opposite direction.

The two atomic event patterns also define a sourceElement parameter, depicting the model element associated with the appearance and disappearance of the violation.

In this solution, an adapter between the Validation framework and the CEP engine generates the atomic events on the changes of the validation query matches. As an alternative, query event patterns could have been defined with the appropriate validation query references. Both alternatives are equivalently suitable to process the validation information. We chose the former solution to rely on the Validation framework instead of relying on the observed model directly and hence separate the concerns.

In the next step, atomic event patterns are combined into a complex event pattern. In Fig. 11, the definition part of the tolerationRange complex event pattern contains the definition of the pattern, i.e., what level of invalidity can be tolerated before the DSE engine gets notified to generate quick fixes. In this specific example, the tolerance threshold is hit after three invalidModel events from the same source are observed after each other. The src formal parameter is a unification directive among the atomic event patterns as defined in Definition 7. The {3} directive is a multiplicity operator (Sect. 4.3.1) applied on the atomic event patterns.

To enable reacting on the complex event pattern, the toleranceLimitReached rule is defined, featuring an executable action (defined in the Xbase language [33]) which invokes the appropriate method of the DSE engine.

### Quick Fix Generation by Design Space Exploration

Quick fixes are generated by a DSE process. This process is configured by (i) objectives to define the desired states of a model a potential quick fix should make reachable; and (ii) transformation rules to define how a model can be transformed.

In this case study, we use only one objective: the model of the CPS should be valid. As shown in Fig. 12, the Viatra-DSE framework supports capturing this objective by reusing the Viatra Queries graph patterns previously defined for validation purposes in a *ModelQueriesHardObjective*. The name suggests that the objective is hard, i.e., it must be satisfied by every potential quick fix candidate. (On the other hand, soft objectives serve as heuristics but are not guaranteed to be satisfied in every case.) The ModelQueryType.NO_MATCH directive suggests that the objective is satisfied if the referred model query

(AvailableGreaterThanTotalCpu, as defined in Figure 10) has no matches at all.

Transformation rules are defined by a left-hand side (LHS) precondition and an action. The former one is again an Viatra Queries graph pattern reference, while the latter one is captured by Java code.

### Execution of the Case Study

**Fig. 13:**
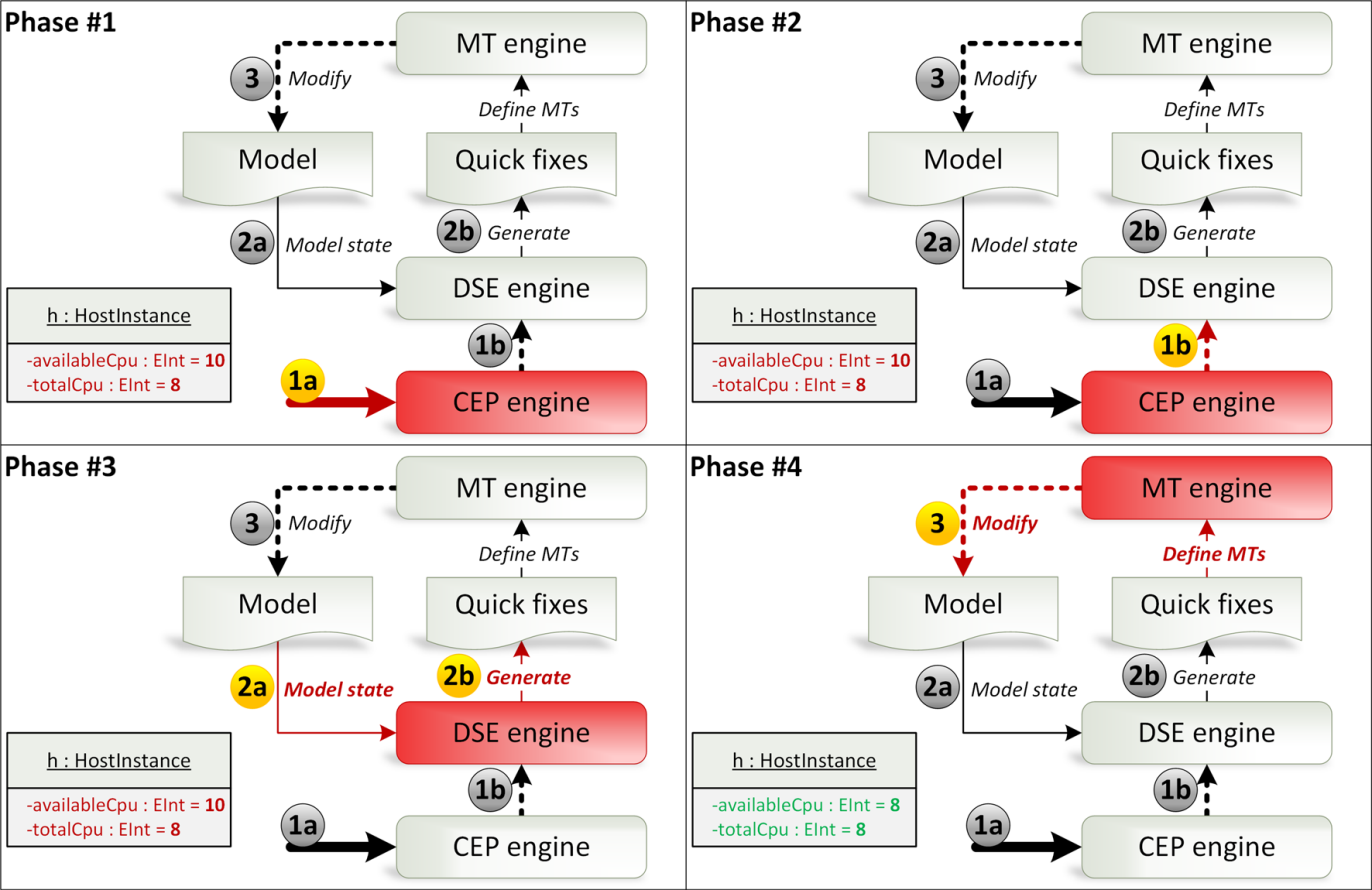
Example execution of the case study

Figure 13 shows an example execution of the case study (in accordance with Fig. 8).

- **Phase 1** An instance of the *HostInstance* type is manipulated by the user, so that its *availableCpu* (10) is greater than its *totalCpu* (8). The Validation framework evaluates the well-formedness query in Fig. 10 and since a new match of the query is found, an *invalidModel* event (Fig. 11) is published on the event stream, and subsequently processed by the CEP engine in **Step 1a**.
- **Phase 2** The model is further modified, but the validation issue still persists and therefore, after every modification an additional *invalidModel* event is published. After observing the third event of this type, the *tolerationRange* complex event pattern is matched and the action defined in the *toleranceLimitReached* rule is executed in **Step 1b**. Consequently, the DSE engine is notified to generate quick fixes.
- **Phase 3** The DSE engine first reads the current state of the model in **Step 2a** and generates the quick fixes in **Step 2b**. Two quick fixes are generated and provided to the user: decreasing the number of available CPUs to the number of the total CPUs (Fig. 12), or the other way around. In this example scenario, the user selects former option.
- **Phase 4** After selecting the quick fix decreasing the number of available CPUs, the model transformation executing this action is passed to the model transformation engine, which applies the transformation on the source model in **Step 3**. Subsequently, the instance of the *HostInstance* type is valid again.

The workflow features multiple *reactive* elements. For example, in **Step 1b** the action notifying the DSE engine is executed as a response to a matched event pattern. Similarly, applying a selected quick fix in **Step 3** is also carried out in a reactive fashion, as a response to the choice of the user. To support this kind of reactive behavior, the EVM (Event-driven Virtual Machine) [13] reactive rule engine is used. Configurations to the components in the case study (validation, CEP, DSE, MT) map to executable EVM programs which are then executed based on the appropriate triggers.

This uniform execution model facilitates easier integration of the components as it reduces the problem of interoperability both in terms of data and control and facilitates process-level integration [8, 72].

### Discussion

**Fig. 14:**
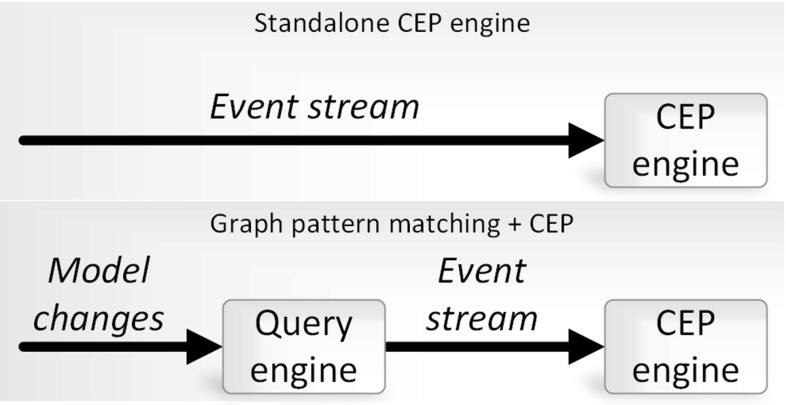
Compared architectures: a standalone CEP engine and one combined with a graph pattern matcher

To assess the reduction of complexity in event patterns, enabled by using compound changes as atomic events, i.e., by using graph pattern matching as an input to complex event processing, we calculate the number of required event patterns for the case study in a theoretical complex event processing architecture without a graph pattern matcher (Fig. 14).

To observe relevant changes in the validity of the model, all seven attributes of the *HostInstance* type (Fig. 10) are required to be monitored by the appropriate elementary change events. These seven elementary/atomic events are later combined into a complex event pattern equivalent to the one in Fig. 11, while the invalid/valid state changes of the model have to be reconstructed based on elementary model changes.

The main challenge here is to identify the combinations that require triggering the DSE engine. The feasibility of such an approach is questionable even in a simple example like the one discussed here. The key advantage of our approach is the shifting of complexity toward a graph pattern matcher that is capable to identify relevant *compound* model changes efficiently, and therefore, enables less efforts on the event modeling side.

The reduced number of event patterns is advantageous from a performance point of view as well, since the size of the automata (i.e., the number of its nodes and transitions) grows linearly along with the atomic event patterns employed in a complex event pattern.

## Evaluation Over Live Models

In this section, we present a case study and use it to assess the usability and the performance limits of the Viatra-CEP framework. This case study carries two important differences as opposed to the running example (Sect. 3). First, this case study features a *materialized, finite but rapidly evolving* live model, instead of a slowly changing infinite streaming model. The live model is intended to capture the prevailing state of a sensor system at run-time. Second, *direct* change events from the underlying model are processed in this case, instead of processing validation information. This motivates the usage of query result change event patterns (Sect. 4.2), instead of atomic event patterns, as the former ones facilitate automated integration with the query engine supervising the underlying model.

The example is based on our preliminary work [27]; and [26], presented earlier at EclipseCon Europe 2012, but without using the framework described in this paper. This section gives a brief overview on the solution and focuses on the results. Relevant parts of the related source can be found in “Appendix 2.” Our previous work [27] discusses the example in more details.^6^

### Gesture Recognition by Streaming Transformations

In the case study, a human body is observed by optical sensors. The stream of data from the sensors (Microsoft Kinect [59] in our case) carry the spatial position of the hands, wrists, knees, etc. This stream is continuously processed and its data are stored in a *live model*, technically, an EMF model maintained via a Java-based API [44]. Every time the optical sensors capture a new frame, the model is updated with the appropriate spatial data. The sensors process 25 frames per second, resulting in 25 model update transactions each second. The complexity of the scenario arises from the frequent changes the model undergoes. Executing model transformations on such a model poses several problems, since it would become obsolete quickly after being loaded into the memory. Moreover, model update transactions affect multiple model elements.

**Fig. 15:**
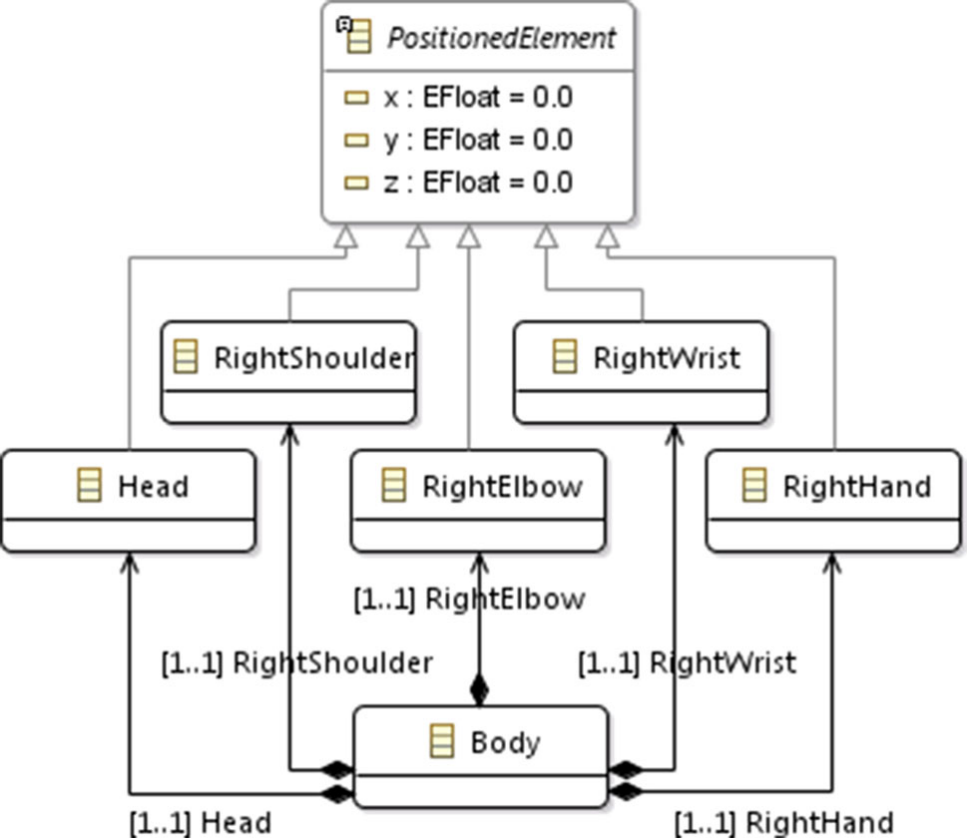
Excerpt from the domain metamodel [44]

Figure 15 shows an excerpt from the domain metamodel [44], containing the head and the right arm. Similar metamodel elements describe the other three limbs of the body.

We aim at recognizing a gesture in order to control a PowerPoint presentation with it. On the recognized gesture, the presentation advances to the next slide; therefore, the gesture is referred to as the *forward gesture*. In [26], there is also a *backward gesture* to move back to the previous slide.

**Fig. 16:**
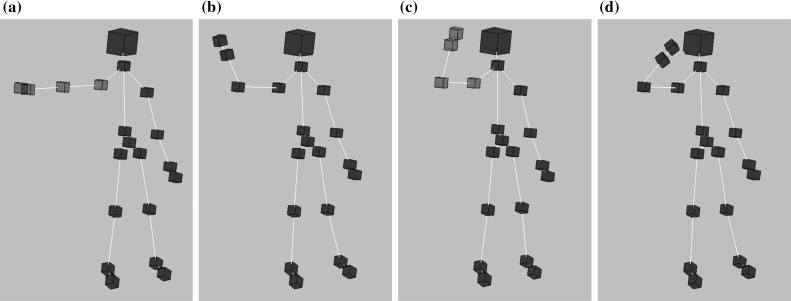
Body postures with the key context of the human body highlighted. **a** Forward start found. **b** Forward start lost. **c** Forward end found. **d** Forward end lost

As illustrated in Fig. 16, the *forward gesture* consists of two postures: the *forward start* and the *forward end*. To recognize the gesture, the series of these two postures needs to be identified. Postures are considered as certain *states* of the body, which are described with a *range* or interval of spatial data. For example, the *forward start* posture is defined by the right arm being approximately stretched to the height of the shoulder. We determine whether the arm is stretched by continuously measuring the angle between the upper and lower arm and smoothing the resulting stream of spatial data by a moving average transformation [16].

Processing a series of postures could be interpreted as a state machine where the states represent postures and transitions are triggered if a body leaves the valid range of the state and enters another. For instance, the body initiates the *forward start* posture by first entering the posture (*forward start found*), then leaving it (*forward start lost*) after a certain amount of time.

### Modeling and Execution

We follow the principles presented in Sect. 6. First, model queries are defined to identify the current state of the model and automatically publish notifications on relevant state changes in the form of atomic event instances. Listing 18a shows the graph pattern depicting the *Forward start* posture, as presented in Listing 16a. The pattern is parameterized with the spatial data of the right arm (consisting of the right hand, the right elbow and the right shoulder); the head; and the body the previous parts belong to. The *Forward start* posture requires a stretched right arm to be detected, but the arm shall not be held higher than head level. The pattern in Listing 18b compares the spatial coordinates of the right hand and the head by their *y* coordinate. This pattern is used as a negative condition in the first pattern.

**Fig. 17:**
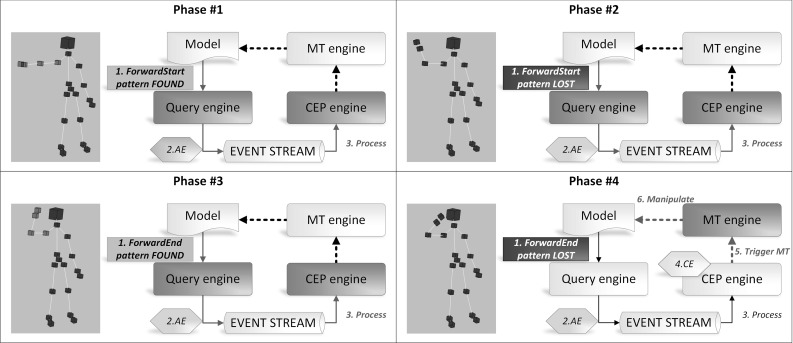
Gesture phases and the execution steps triggered

As opposed to the solution in Sect. 6, atomic event instances now represent direct model changes, and not derived validation information. Therefore, query result change event patterns (Definition 1) are used as atomic event patterns as they can refer to Viatra Queries patterns directly. This unique feature of our language aims to seamlessly integrate a language for graph patterns with a language for event patterns.

Query result change events in Listing 19 are parameterized with a Body parameter. This enables collecting atomic events per body, i.e., to distinguish between atomic events based on their source.

Finally, the complex event patterns and related actions with rules are defined, as presented in Listing 20.

### Execution of the Case Study Example

Figure 17 summarizes the execution steps triggered by four consecutive snapshots of the forward gesture.

- **Phase #1.** The *ForwardStart* pattern (Listing 18a) is found (Step *1*) in the model by the query engine. This results in a new tuple of model elements as a match set, whose data are wrapped into an atomic event by the query engine and passed to the event stream (Step *2*). The CEP engine processes the atomic event instance (Step *3*) and updates the complex event patterns. As the *ForwardGesture* (Listing 20a) complex event pattern is not matched yet, this phase ends here.
- **Phase #2 and #3.** In the next phase, we detect that a match of the *ForwardStart* pattern is lost. The same steps are executed as above, only this time an atomic event of type *ForwardStartLost* is published on the event stream and processed by the CEP engine. In Phase #3, a *ForwardEndFound* atomic event is identified and placed on the stream.
- **Phase #4.** The *ForwardEnd* pattern is lost and a *ForwardEndLost* atomic event is published on the event stream consequently. Now, there will be additional steps triggered after Step *3*. After having processed the *ForwardEndLost* atomic event, the CEP engine matches the *ForwardGesture* complex event pattern (Step *4*) and triggers the execution of the associated rule (Listing 20b) by triggering the model transformation defined in the rule (Step *5*). The MT engine identifies the activated transformation rule and executes it (Step *6*).

### Evaluation

To estimate the performance and scalability of our tool, we had to design a semi-synthetic benchmark based on the case study. The reason for this is that Microsoft Kinect can only detect at most two bodies, and the refresh rate is a fixed 25 frames per second (FPS), which is easily processed by our CEP engine.

**Table 3:** Throughput and the highest processing speed

Body count	Complex event throughput	Atomic event throughput	Atomic events in the model	Processing speed
#	[1/s]	[1/s]	[1/cycle]	[x 25 FPS]
1	69,041	414,248	6	69,041
2	63,458	380,749	12	31,729
4	66,094	396,562	24	16,523
8	41,907	251,442	48	5,238
16	35,003	210,017	96	2,188
24	24,220	145,322	144	1,009
25	20,611	123,664	150	0,824

#### Evaluation Setup

The core of the simulation is a previously recorded real execution sequence in which the right arm is rotated. A full arm cycle consists of 12 positions, i.e., 12 frames. Every cycle yields exactly one *Forward gesture* (Fig. 16) composed of the sequence of 4 atomic events; and every cycle also yields two atomic events considered as noise. This makes 6 atomic events generated for each cycle.

Our simulations aim at stress testing our CEP prototype, which is carried out by multiplying this sequence along a different number of bodies in the model. This part of the benchmark scenario is artificial in the sense that Kinect can handle at most two bodies, but the actual positions of the bodies remain realistic.

After starting the simulations, we primarily measure the *number of detected complex events per second*. From this rate, we calculate the effective processing rate (i.e., the theoretical upper limit) of the CEP engine measured in *frames per second* (FPS). This value is compared to the original FPS rate of the Kinect sensor. We continue increasing the number of bodies up to the point when the processing rate is greater than the recording rate.

#### Summary of Performance Results

Even though there are many approaches using Kinect for gesture recognition and other similar tasks, these approaches either lack the explicit live/runtime model representation (thus prohibiting graph reasoning) or the assessed performance aspects (such as precision, recall or lift factor used in machine learning [48]) do not reflect runtime performance of the engine. [30, 49, 75] We identified, therefore, relevant static and dynamic metrics in order to evaluate our work, and that from the aspect of scalability in the first place.

Table 3 summarizes our results. Rows represent the individual measurements with respect to the increasing number of bodies *Body count*. The next two columns present the throughput of *complex events* (1/s) and *atomic events* (1/s), respectively. The latter is calculated from the former, since for every complex event to be detected, 6 atomic events are observed (as discussed above). The number of *atomic events in the model* denotes how many atomic events are triggered by elementary or compound model changes *per cycle*, i.e., while the right arm makes a circle. This is the number of atomic events *required* to be processed in order to achieve the frames per second (FPS) ratio the Kinect sensors work with. Finally, *processing speed* summarizes the FPS of our prototype compared to the basic FPS value of Kinect (25). This value is calculated as the ratio of the *Atomic event throughput* and the *Atomic events in the model*. This ratio is acceptable if it is above 1; otherwise, the processing rate of complex events falls short to the data production rate of the Kinect sensor.

As a summary, our measurements show that our approach scales up to 24 bodies in the model (the lowest processing speed above 1) at $25\times 1.009$ FPS. In order to interpret this value, we need to recall that one body consists of 20 control points each of them containing 6 attributes (see *PositionedElements* in Fig. 15), from which 2 are actually modified in the simulations. Therefore, for each body, 40 elementary model changes are triggered in every frame (assuming that the limbs are not reattached to different bodies).

Handling 24 bodies at a rate of $25\times 1.009$ FPS yields approximately 24000 complex events per second, which implies 150.000 atomic events per second. (Measurements were carried out using a 2.9 GHz CPU.) [35] defines the linear scalability limit of the Esper platform in 500.000 events per second, which is in the same order of magnitude as Viatra-CEP. Considering the performance optimization of our tooling being a future work, we conclude that our proof-of-concept implementation offers promising performance and scalability.

It should be noted, however, that due to the rather simple movement profile (only a few coordinates are manipulated), the results cannot be trivially extrapolated for data streams of real Kinect devices.

#### Usability Comparison

Over the course of implementing of the case study, we also observed the usability and productivity aspects of our approach. Table 4 summarizes our findings.

**Table 4:** Comparison of the two approaches

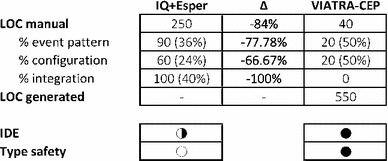	

*Reduced amount of source code.*  The Viatra-CEP framework enables a model-driven approach to streaming transformations compared to our previous work [26]. As code generation is one of the traditionally emphasized highlights of such a paradigm shift [53], our approach significantly reduces the amount of source code required for these scenarios. Compared to the previous version of this complex gesture recognition case study, we observed a decrease of 84 % in terms of *manually written lines of code (LOC)* used in this example. The LOC decreased significantly in event pattern and rule definitions (around 78 %) and in the configuration tasks (around 67 %), such as setting up the engine, wiring event pattern and definitions. The manually written integration (glue) code between the graph pattern matcher and the CEP engine completely disappeared, as Viatra-CEP supports integration with Viatra Queries out-of-the-box. This significant reduction of source code is enabled by the powerful DSL, from which approximately 550 lines of code are *generated*. All of these are significant software engineering benefits.

*Automated application management.* Our Eclipse-based prototype IDE hides most application life cycle management. At design time, a rich textual editor is provided to the user to model event patterns with support for syntax highlight, context-sensitive assistance and validation. The IDE also makes use of Eclipse-related facilities, such as automated project metadata handling, dedicated builder facilities, and project and model creation wizards. The graph patterns are modeled using the IDE of Viatra Queries, while type safety over the disparate domains of graph pattern matching and event processing is also maintained and hidden from the user. This way, the level of our tooling is more comparable to the industrial Drools Fusion framework while Esper still does not provide an IDE for modeling complex events.

## Related Work

In this section, we give an overview of various approaches related to our work. Table 5 presents an overview of the state of the art considered at this place. We compared our work to approaches and tools from the domains of *Graph reasoning* and *Complex event processing*. As a general takeaway, our contributions include the following:

- combined semantics for graph reasoning and complex event processing;
- extending the streaming transformation concept to live models and models@run.time;
- support for reactive transformations by reusing the concept of change-driven transformations (CDT).

**Table 5:** Overview of the related work with respect to *Graph reasoning* and *Complex event processing* (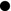/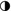/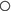: fully/partially/not supported; N/A: not applicable)

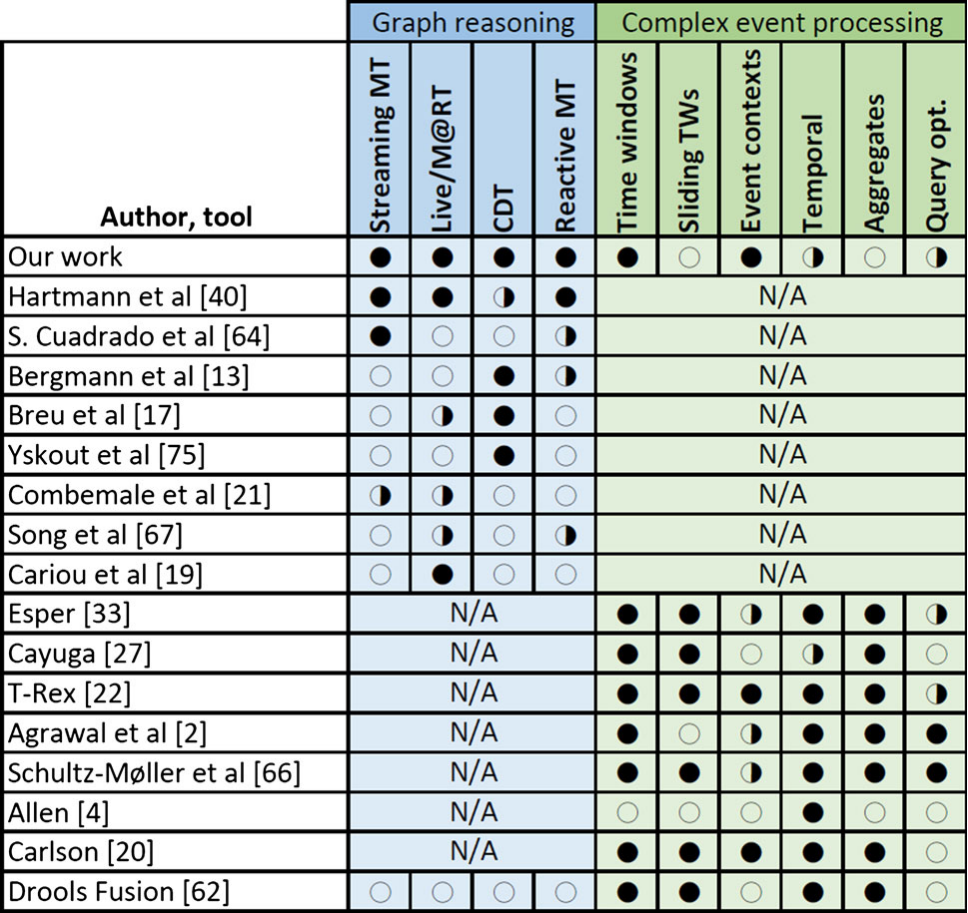	

### Graph Reasoning

Hartmann et al [41] present a distributed models@run.time approach, combining ideas from reactive programming, peer-to-peer distribution, and large- models@run.time. Similarly to [65], models are defined as observable streams of model fragments. Fragments are distributed between nodes in a peer-to-peer on-demand fashion which eliminates the need for passing around full models. As compared to our approach, the authors not employ event-based paradigms, but view runtime models themselves as continuous streams.

Sánchez Cuadrado and De Lara [65] present streaming transformations working on a stream of model fragments and elements. In contrast to this technique, our approach leverages derived information regarding the model in the form of change events, which decouples the execution from the actual model. Consequently, some of the issues discussed in by the authors (e.g., dealing with references among model elements and transformation scheduling) are not present in our case. We also extended the streaming transformation concept to *live and runtime models*.

To efficiently capture arbitrarily compound structural changes, we reuse the techniques of change-driven transformations (CDT), previously presented by Bergmann et al. [14], Yskout et al [76] and in [9]. As a main advantage over these techniques, our technique allows detecting more complex structures of compound changes and identify compound changes on a higher granularity.

A formal foundation of infinite models was introduced by Combemale et al. [22] by redefining OCL operators over infinite collections. This is complementary problem as the models themselves are finite in our case, but their lifeline is infinite due to the model changes. Song et al. introduced incremental QVT transformations [68] for runtime models. However, these techniques primarily focus on obtaining a faithful model of the running system, while they do not consider event streams or complex event processing over live models.

Cariou et al. [20] investigate the possibilities of adapting directly executed models for validation purposes. The adaptation logic is event-driven, however relies on atomic events only. By processing complex events, a more complex adaptation logic could be achieved. The motivating example discusses fail-stop behavior on model deviations. This behavior can be enhanced, for example, by considering events that handle, compensate these deviations and are acceptable if observed in a certain time window. In our current paper, we showed that a live model-based approach can be feasible for detecting validation issues. Additionally, we provided a DSE-based approach for handling validation issues in a semi-automated way.

### Complex Event Processing

By comparing the Vepl language to the state of the art of complex event processing, some of the obvious limitations become clear. This is due to the fact that we use CEP as a supporting technique and shift the complexity toward the graph reasoning part of our approach. As a general limitation, the Vepl language does not feature rich aggregate functions and sliding time windows, as the typical use cases of streaming model transformations do not necessitate such features.

Esper [34] is an open-source event processing framework. It has been employed in our preliminary work [26], presented at the EclipseCon Europe 2012. Despite being a high-end CEP engine concerning its performance and the descriptive power of its language, supporting the scenarios presented in this paper is infeasible. An additional drawback of the platform is the lack of an integrated modeling environment, which makes integration with design/development processes cumbersome. Esper has a rich event processing language, which, as opposed to Vepl, allows defining aggregates and sliding windows as well. Event contexts, however, are not modeled explicitly, but worked around by stream manipulations.

Cayuga [28] is a non-deterministic infinite automaton-based event processing environment. Instead of a finite alphabet, it allows arbitrary inputs and filters them according to user-defined constraints.

The T-Rex [23] event processing middleware and its event pattern language, TESLA, combines expressiveness and efficiency. Similarly to our approach, the authors choose an incremental event processing strategy batch-like solutions in order to reduce latency in the processing. The pattern language provides rich semantics to define complex event patterns. TESLA uses *consumption policies* [24] to model event contexts. Static and dynamic constraints correspond to check expressions of Vepl, but with support for aggregates and sliding window specifications.

Agrawal et al. [2] propose a model for event query evaluation, based on the combination of a non-deterministic finite automata and a match buffer. The latter feature is a main difference as opposed to our formalism that allows efficient temporal reasoning over structures such as the Kleene-plus operator. Although in our approach, both the Kleene-star and the Kleene-plus operators are supported, they only allow a subset of temporal patterns to be defined. The approach also handling *active time windows*, i.e., time windows that can expire based on a physical clock, as opposed to passive time windows whose expiration is checked upon moving an associated token. The authors claim that even though active time windows require more formulas to be evaluated at runtime, they also prune non-viable traces earlier, resulting in better performance measures.

Schultz-Møller et al. [67] address the problem of query optimization in CEP systems, motivated by the similar scenarios in relational database systems. Queries are rewritten and optimized with respect to cost models of complex event operators, but also considering distributed execution among multiple computation nodes. The approach shows significant similarities to the one presented in this paper, both in terms of the high-level modeling language and the execution semantics. Even though the modeling language is more SQL-like (as opposed to Vepl), the set of operators and the simple, yet expressive automaton-based formalism enable capturing essentially the same subset of complex event patterns.

Allen’s interval algebra [4] is a foundational work on temporal relations of intervals. Our work implements a subset of Allen’s algebra, omitting the refined level of parallel relations, but extending the temporal timing aspect with absolute (physical or logical) timing. The terminology and concepts used in our work are mainly influenced by Carlson’s event detection algebra (EDA) [21], although we augment our approach by a domain-specific and extensible modeling language which hides the elaborate details of the algebra from the user. We also employ optimization steps as event patterns are compiled into automata.

Drools Fusion [63] is an open-source complex event processing platform. As a part of the larger JBoss/Drools ecosystem, Drools Fusion builds on top of a business rules management system (BRMS). Although it can be considered as a technique similar to ours, that integrates graph reasoning and complex event processing, the Drools stack focuses more on business processes. Our framework builds on a more general model management framework, and generally targets system/software engineering scenarios. Consequently, Drools Fusion lacks the support for several model transformation scenarios.

### Other Related Approaches

Hinze and Voisard [46] introduce the EVA language as an extensive semantic foundation for translating and unifying various event description and processing languages. In order to ensure conformance with other algebras, the language generalizes the common event algebraic operators. By comparing the Esper and the RTEC platforms, Elias and Alexander [3] draw the conclusions that translating two significantly different event languages, although not a trivial task, is certainly possible.

Our previous work [25] presents a complex event description language (CEDL) which can be considered the preliminary version of Vepl. Our current DSL, however, shows significant improvements to CEDL by introducing query result change events and executable actions (for example model transformations). Another important difference is the execution model of the two languages. While our previous work was mapped and executed on top of the Esper [34] platform, our current DSL is a part of a whole complex event processing platform of its own.

Deshayes and Mens [30] use statecharts to model complex gestures and validate their approach using a similar setting as the one presented in Sect. 7. The authors conclude that statechart, as a high-level modeling language, reduces accidental complexity. Our approach further improves this aspect by allowing hierarchical event structures to be defined and used as triggers for executable actions (such as model transformations).

Schmidt et al. [66] conclude that RETE networks [39] are not suitable for complex event processing as described by Luckham [58]. To overcome its limitations, the authors augment RETE networks with dedicated event processing nodes which keeps the event processing and fast reasoning concerns separated. As the Viatra-CEP framework relies on the Viatra Queries engine featuring a RETE network, it is an interesting research direction how the event processing capabilities of Viatra-CEP can contribute to the RETE-based pattern matching features of Viatra Queries.

Esteves Veríssimo et al. [36] discuss how conventional non-event-driven architectures can be extended in order to publish events for diagnostic purposes. The authors bring motivating examples from the domain of monitoring financial information systems, which is also a typical domain for employing streaming transformations over live models.

Apache Camel [5] is an integration framework supporting complex event processing. The framework natively supports defining and detecting event patterns using the Esper platform. Alternatively, its typesafe DSL for Java, Scala and Groovy provided by Camel RX [6] enables defining and processing events as natural collection-like structures.

## Conclusions and Future Work

In this paper, we presented a novel approach for streaming model transformations by combining change-driven model transformations (CDT) and complex event processing (CEP). Thorough algebraic foundations for the required event processing language have been provided along with a prototype implementation, the Viatra Event Processing Language (Vepl). The static structure, the operator semantics and the execution semantics have been formally defined and the completeness and soundness of the execution has been proved.

We also presented an advanced MDE scenario in which the underlying model cannot be materialized and is available as an infinite stream of model elements (Sect. 3), carrying well-formedness information about a domain instance model. As opposed to the case study in Sect. 7, the complexity of the problem does not arise from a rapidly changing live model, but due to integration scenarios between different elements of the heterogeneous workflow. It has been shown that the greater Viatra framework provides easy data-, control- and workflow-integration facilities [8] due to the common runtime environment, EVM. The modeling process is also enhanced by the Viatra Queries framework, as components of the Viatra framework can reuse shared libraries of model queries for various objectives (such as, defining query event patterns, or transformation rules for the design space exploration process).

*Future work* As general future work, we plan to apply our framework in various domains, as we find this the key to improve the Vepl language in a useful and reasonable way. *Smart cyber-physical systems* in open and dynamically changing environments give rise to modeling problems similar to those discussed in this paper. [29] Models undergoing rapid changes and infinite non-materializable models are typical in this domain and advanced techniques with proper theoretical foundations and tooling are required to address the related modeling scenarios. These systems also motivate various *distributed* event processing scenarios. We plan to extend our framework in this direction, with special focus on settings featuring computationally constrained event processing resources, as presented in our works [62] and [11]. The approach of Schultz-Møller et al. [67] gives a feasible starting point because of the significant similarities with our approach in terms of event representation and execution semantics.

We plan to further investigate the execution formalisms suitable for event processing. Apart from the widely used DFA- and NFA-based approaches, DEVS [71] can be considered as an alternative, because of its explicit timing semantics. Enhanced RETE algorithms [66] can serve as models for complex event-based reactive execution. We envision a hybrid execution formalism which allows choosing the most suitable representation based on the given event processing problem during compilation time.

As mentioned in Sect. 3, model inconsistency tolerance rules (in our case depicted by complex event patterns) can be typically extracted from design processes and inter-model dependency models. Due to their typically inter-domain nature, model inconsistencies are hard to characterize and even harder to tackle in an efficient automated way. [45] We foresee inconsistency tolerance as a key feature to an efficient and well-scalable inconsistency management framework. Our ongoing work focuses on the formalization of an *underlying consistency algebra* which the tolerance rules conform to and which facilitates the automated extraction of such rules.

As a primary direction for the more technical future work, we plan several enhancements to the tooling, for example a *visual debugger* for observing the runtime behavior of event patterns at run-time. The thorough *performance assessment* of the framework is an ongoing work for which we use the Linear Road benchmark [7]. We plan to address the scalability of our tool by investigating alternatives for make it partitioned [64] and distributed [52] [69].

## Figures and Tables

**Fig. 18:**
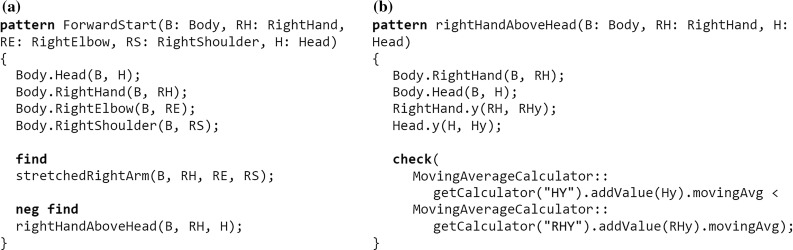
Viatra queries for the example in Sect. 7. **a** ForwardStart posture. **b** rightHandAboveHead

**Fig. 19:**
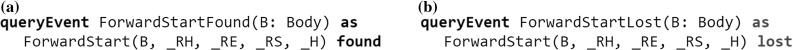
Viatra-CEP patterns reusing the queries of Fig. 18. **a** ForwardStartFound event. **b** ForwardStartLost event

**Fig. 20:**
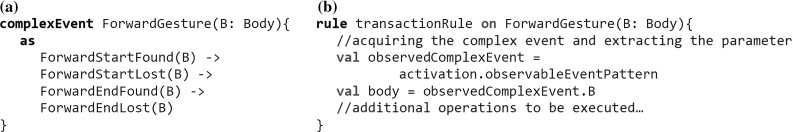
Complex event pattern as a trigger for a transformation rule. **a** ForwardGesture complex event pattern. **b** A streaming transformation rule

## Appendices

### Appendix 1: Propositions and Proofs

#### Associativity of Vepl Operators

##### Proposition 4

**Associativity of the binary operators** All the binary operators of the algebra are associative by definition. Formally:

- $$E_1^n \models \mathtt {fol}(c_1, c_2, c_3) \Leftrightarrow E_1^n \models \mathtt {fol}(\mathtt {fol}(c_1, c_2), c_3) \Leftrightarrow E_1^n \models \mathtt {fol}(c_1, \mathtt {fol}(c_2, c_3))$$.
- $$E_1^n \models \mathtt {or}(c_1, c_2, c_3) \Leftrightarrow E_1^n \models \mathtt {or}(\mathtt {or}(c_1, c_2), c_3) \Leftrightarrow E_1^n \models \mathtt {or}(c_1, \mathtt {or}(c_2, c_3))$$.
- $$E_1^n \models \mathtt {and}(c_1, c_2, c_3) \Leftrightarrow E_1^n \models \mathtt {and}(\mathtt {and}(c_1, c_2), c_3) \Leftrightarrow E_1^n \models \mathtt {and}(c_1, \mathtt {and}(c_2, c_3))$$. $$\square $$

##### Proof

*Associativity of the* $\mathtt {fol}$ *operator* $E_1^n \models \mathtt {fol}(c_1, c_2, c_3)$ iff $E_1^n \models c_1 ~\wedge ~ E_1^n \models c_2 ~\wedge ~ E_1^n \models c_3$, and $c_1.\tau \le c_2.\tau \le c_3.\tau$.

- $E_1^n \models c_1 ~\wedge ~ E_1^n \models c_2$ and $c_1.\tau \le c_2.\tau$ iff $E_1^n \models \mathtt {fol}(c_1, c_2)$ and $\mathtt {fol}(c_1, c_2).\tau = c_2.\tau$ (Due to Definition 9.)
- $E_1^n \models c_2 ~\wedge ~ E_1^n \models c_3$ and $c_2.\tau \le c_3.\tau$ iff $E_1^n \models \mathtt {fol}(c_2, c_3)$ and $\mathtt {fol}(c_2, c_3).\tau = c_3.\tau$ (Due to Definition 9.)

Consequences:

- $$E_1^n \models \mathtt {fol}(c_1, c_2) ~\wedge ~ E_1^n \models c_3)$$ iff $$E_1^n \models \mathtt {fol}(\mathtt {fol}(c_1, c_2), c_3)$$
- $$E_1^n \models \mathtt {fol}(c_2, c_3) ~\wedge ~ E_1^n \models c_1)$$ iff $$E_1^n \models \mathtt {fol}(c_1, \mathtt {fol}(c_2, c_3))$$ $$\square $$

##### Proof

*Associativity of the* $\mathtt {or}$ *operator* $E_1^n \models \mathtt {or}(c_1, c_2, c_3)$ iff $E_1^n \models c_1 ~\vee ~ E_1^n \models c_2 ~\vee ~ E_1^n \models c_3$.

$E_1^n \models c_1 \Rightarrow \mathtt {or}(c_1, \mathtt {or}(c_2, c_3))$ immediately without evaluating the right-side operand; and $E_1^n \models c_1 \Rightarrow \mathtt {or}(\mathtt {or}(c_1, c_2), c_3)$ by evaluating the left-side operand.

$E_1^n \models c_2 \Rightarrow \mathtt {or}(c_1, \mathtt {or}(c_2, c_3))$ by evaluating the right-side operand; and $E_1^n \models c_1 \Rightarrow \mathtt {or}(\mathtt {or}(c_1, c_2), c_3)$ by evaluating the left-side operand.

$E_1^n \models c_3 \Rightarrow \mathtt {or}(c_1, \mathtt {or}(c_2, c_3))$ immediately without evaluating the left-side operand; and $E_1^n \models c_1 \Rightarrow \mathtt {or}(\mathtt {or}(c_1, c_2), c_3)$ by evaluating the right-side operand. $\square$

##### Proof

*Associativity of the* $\mathtt {and}$ *operator* $E_1^n \models \mathtt {and}(c_1, c_2, c_3)$ iff $E_1^n \models c_1 ~\wedge ~ E_1^n \models c_2 ~\wedge ~ E_1^n \models c_3$.

- $$E_1^n \models c_1 ~\wedge ~ E_1^n \models c_2 ~\wedge ~ E_1^n \models c_3 \Rightarrow E_1^n \models \mathtt {and}(c_1, c_2) ~\wedge ~ E_1^n \models c_3 \Rightarrow E_1^n \models \mathtt {and}(\mathtt {and}(c_1, c_2), c_3)$$.
- $$E_1^n \models c_1 ~\wedge ~ E_1^n \models c_2 ~\wedge ~ E_1^n \models c_3 \Rightarrow E_1^n \models c_1 ~\wedge ~ \mathtt {and}(c_2, c_3) \Rightarrow E_1^n \models \mathtt {and}(c_1, \mathtt {and}(c_2, c_3))$$. $$\square $$

#### Commutativity of Vepl Operators

##### Proposition 5

**Commutativity of the binary operators** Operator $\mathtt {fol}$ is not commutative, while $\mathtt {or}$ and $\mathtt {and}$ are commutative. Formally:

- $$E_1^n \models \mathtt {or}(c_1, c_2) \Leftrightarrow E_1^n \models \mathtt {or}(c_2, c_1)$$.
- $$E_1^n \models \mathtt {and}(c_1, c_2) \Leftrightarrow E_1^n \models \mathtt {and}(c_2, c_1)$$.$$\square $$

##### Proof

*Non-commutativity of the* $\mathtt {fol}$ *operator* $E_1^n \models \mathtt {fol}(c_1, c_2)$ iff $E_1^n \nvDash \mathtt {fol}(c_2, c_1)$, because of the order preserving property of the operator. $\square$

##### Proof

*Commutativity of the* $\mathtt {or}$ *operator* $E_1^n \models \mathtt {or}(c_1, c_2)$ iff either $c_1$ or $c_2$ is matched. Therefore $E_1^n \models \mathtt {or}(c_2, c_1)$. $\square$

##### Proof

*Commutativity of the* $\mathtt {and}$ *operator* $E_1^n \models \mathtt {and}(c_1, c_2)$ iff $c_1$ and $c_2$ are matched. Due to the not order preserving nature of the operator, it can be also stated that $E_1^n \models \mathtt {and}(c_2, c_1)$. $\square$

#### Distributivity of Vepl Operators

##### Proposition 6

**Distributivity of the binary operators** Operators $\mathtt {fol}$ and $\mathtt {and}$ are distributive over the $\mathtt {or}$ operator. That is, both operators are both left- and right-distributive over $\mathtt {or}$. Formally:

- $$E_1^n \models \mathtt {fol}(c_1, \mathtt {or}(c_2, c_3)) \Leftrightarrow E_1^n \models \mathtt {or}(\mathtt {fol}(c_1, c_2), \mathtt {fol}(c_1, c_3))$$.
- $$E_1^n \models \mathtt {fol}(\mathtt {or}(c_1, c_2), c_3) \Leftrightarrow E_1^n \models \mathtt {or}(\mathtt {fol}(c_1, c_3), \mathtt {fol}(c_2, c_3))$$.
- $$E_1^n \models \mathtt {and}(c_1, \mathtt {or}(c_2, c_3)) \Leftrightarrow E_1^n \models \mathtt {or}(\mathtt {and}(c_1, c_2), \mathtt {and}(c_1, c_3))$$.
- $$E_1^n \models \mathtt {and}(\mathtt {or}(c_1, c_2), c_3) \Leftrightarrow E_1^n \models \mathtt {or}(\mathtt {and}(c_1, c_3), \mathtt {and}(c_2, c_3))$$. $$\square $$

To prove the proposition, both the left-distributivity and the right-distributivity must be proved.

##### Proof

*Left-distributivity of* $\mathtt {fol}$ *over* $\mathtt {or}\, E_1^n \models \mathtt {fol}(c_1, \mathtt {or}(c_2, c_3))$ iff

- $E_1^n \models c_1$, and following that, either
- $E_1^n \models c_2$, and consequently $E_1^n \models \mathtt {fol}(c_1, c_2)$, or
- $$E_1^n \models c_3$$, and consequently $$E_1^n \models \mathtt {fol}(c_1, c_3)$$.

Which is equivalent to $E_1^n \models \mathtt {or}(\mathtt {fol}(c_1, c_2), \mathtt {fol}(c_1, c_3))$. $\square$

##### Proof

*Right-distributivity of* $\mathtt {fol}$ *over* $\mathtt {or}\,E_1^n \models \mathtt {fol}(\mathtt {or}(c_1, c_2), c_3)$ iff either

- $$E_1^n \models c_1$$, or
- $E_1^n \models c_2$, and following that,
- $$E_1^n \models c_3$$.

This means,

- $$E_1^n \models \mathtt {fol}(c_1, c_3)$$, or
- $$E_1^n \models \mathtt {fol}(c_2, c_3)$$, respectively.

This is equivalent to $E_1^n \models \mathtt {or}(\mathtt {fol}(c_1, c_3), \mathtt {fol}(c_2, c_3))$. $\square$

##### Proof

*Left-distributivity of* $\mathtt {and}$ *over* $\mathtt {or}\, E_1^n \models \mathtt {or}(\mathtt {and}(c_1, c_2), \mathtt {and}(c_1, c_3))$ iff $E_1^n \models \mathtt {or}(\mathtt {or}(\mathtt {fol}(c_1, c_2), \mathtt {fol}(c_2, c_1)), \mathtt {or}(\mathtt {fol}(c_1, c_3), \mathtt {fol}(c_3, c_1)))$, due to the definition of the $\mathtt {and}$ operator, and consequently, $E_1^n \models \mathtt {or}\; (\mathtt {fol}\;(c_1, c_2), \mathtt {fol}(c_2, c_1), \mathtt {fol}(c_1, c_3), \mathtt {fol}(c_3, c_1))$, due to the associative nature of the $\mathtt {or}$ operator. This formula can be satisfied iff

- $$E_1^n \models c_1$$, and
- $$E_1^n \models c_2 ~\vee ~ E_1^n \models c_3$$.

This is equivalent to $E_1^n \models \mathtt {and}(c_1, \mathtt {or}(c_2, c_3))$. $\square$

##### Proof

*Right-distributivity of* $\mathtt {and}$ *over* $\mathtt {or}$ Due to the commutative nature of the $\mathtt {and}$ operator (Proposition 5), the right-distributivity is implied by the left-distributivity. $\square$

##### Proof

*Distributivity of the* $\mathtt {fol}$ *and* $\mathtt {and}$ *operators over* $\mathtt {or}$ Both the $\mathtt {fol}$ and $\mathtt {and}$ are both left- and right-distributive over $\mathtt {or}$ and therefore, both operators are distributive over $\mathtt {or}$. $\square$

#### Transitivity of Vepl Operators

##### Proposition 7

**Transitivity of the binary operators** Operators $\mathtt {fol}$ and $\mathtt {and}$ are transitive, while the $\mathtt {or}$ operator is not transitive. Formally:

- $$E_1^n \models \mathtt {fol}(c_1, c_2) ~\wedge ~ E_1^n \models \mathtt {fol}(c_2, c_3) \Rightarrow E_1^n \models \mathtt {fol}(c_1, c_3)$$.
- $$E_1^n \models \mathtt {and}(c_1, c_2) ~\wedge ~ E_1^n \models \mathtt {and}(c_2, c_3) \Rightarrow E_1^n \models \mathtt {and}(c_1, c_3)$$. $$\square $$

##### Proof

*Transitivity of the* $\mathtt {fol}$ *operator* $E_1^n \models \mathtt {fol}(c_1, c_2) ~\wedge ~ E_1^n \models \mathtt {fol}(c_2, c_3)$ iff $E_1^n \models c_1 ~\wedge ~ E_1^n \models c_2 ~\wedge ~ E_1^n \models c_3$, and the ordering relation is preserved. Therefore: $E_1^n \models \mathtt {fol}(c_1, c_3)$. $\square$

##### Proof

*Transitivity of the* $\mathtt {and}$ *operator* $E_1^n \models \mathtt {and}(c_1, c_2) ~\wedge ~ E_1^n \models \mathtt {and}(c_2, c_3)$ iff

- $$E_1^n \models \mathtt {or}(\mathtt {fol}(c_1, c_2), \mathtt {fol}(c_2, c_1))$$, and (1)
- $$E_1^n \models \mathtt {or}(\mathtt {fol}(c_2, c_3), \mathtt {fol}(c_3, c_2))$$, respectively. (2)

Both cases can be satisfied by any of the two arguments of the $\mathtt {or}$ operator. Transitivity must be shown for the four cases generated by combining the two cases of (1) and (2). That is, for every case, $E_1^n \models \mathtt {and}(c_1, c_3)$ must be shown. (Following the notation of 1.1 being $\mathtt {fol}(c_1, c_2)$, 1.2 being $\mathtt {fol}(c_2, c_1)$, 2.1 being $\mathtt {fol}(c_2, c_3)$ and 2.2 being $\mathtt {fol}(c_3, c_2)$, respectively.)

- **1.1 with 2.1** $E_1^n \models \mathtt {fol}(c_1, c_2) ~\wedge ~ E_1^n \models \mathtt {fol}(c_2, c_3)$ iff $E_1^n \models c_1 ~\wedge ~ E_1^n \models c_2 ~\wedge ~ E_1^n \models c_3$, due to the transitivity of the $\mathtt {fol}$ operator, which implies $E_1^n \models \mathtt {and}(c_1, c_3)$.
- **1.2 with 2.2** For similar reasons and due the commutative nature of the $\mathtt {and}$ operator, $E_1^n \models \mathtt {fol}(c_2, c_1) ~\wedge ~ E_1^n \models \mathtt {fol}(c_3, c_2)$ iff $E_1^n \models \mathtt {and}(c_1, c_3)$.
- **1.2 with 2.1** $E_1^n \models \mathtt {fol}(c_1, c_2) ~\wedge ~ E_1^n \models \mathtt {fol}(c_3, c_2)$ iff $c_1$ and $c_3$ precede $c_2$, but the order of the former two is not decided, that is $E_1^n \models \mathtt {fol}(c_1, c_3, c_2) ~\vee ~ E_1^n \models \mathtt {fol}(c_3, c_1, c_2)$. Due to the left-associativity of the $\mathtt {fol}$ operator (Proposition 4), this can be rewritten into $E_1^n \models \mathtt {fol}(\mathtt {fol}(c_1, c_3), c_2) ~\vee ~ E_1^n \models \mathtt {fol}(\mathtt {fol}(c_3, c_1), c_2)$, or more formally: $E_1^n \models \mathtt {or}(\mathtt {fol}(\mathtt {fol}(c_1, c_3), c_2), \mathtt {fol}(\mathtt {fol}(c_3, c_1), c_2))$. Due to the right-distributive property of $\mathtt {fol}$ over $\mathtt {or}$ (Proposition 6), this yields $E_1^n \models \mathtt {fol}(\mathtt {or}(\mathtt {fol}(c_1, c_3), \mathtt {fol}(c_3, c_1)), c_2)$, which can be rewritten as $E_1^n \models \mathtt {fol}(\mathtt {and}(c_1, c_3), c_2)$, which includes $E_1^n \models \mathtt {and}(c_1, c_3)$. $\square$
- **2.1 with 2.2** Similarly, $E_1^n \models \mathtt {fol}(c_2, c_1) ~\wedge ~ E_1^n \models \mathtt {fol}(c_2, c_3)$ iff $c_2$ precedes $c_1$ and $c_3$, but the order of the latter two is not decided, that is $E_1^n \models \mathtt {fol}(c_2, c_1, c_3) ~\vee ~ E_1^n \models \mathtt {fol}(c_2, c_3, c_1)$. Due to the right-associativity of the $\mathtt {fol}$ operator (Proposition 4), this can be rewritten into $E_1^n \models \mathtt {fol}(c_2, \mathtt {fol}(c_1, c_3)) ~\vee ~ E_1^n \models \mathtt {fol}(c_2, \mathtt {fol}(c_3, c_1))$, or more formally: $E_1^n \models \mathtt {or}(\mathtt {fol}(c_2, \mathtt {fol}(c_1, c_3)), \mathtt {fol}(c_2, \mathtt {fol}(c_3, c_1)))$. Due to the left-distributive property of $\mathtt {fol}$ over $\mathtt {or}$ (Proposition 6), this yields $E_1^n \models \mathtt {fol}(c_2, \mathtt {or}(\mathtt {fol}(c_1, c_3), \mathtt {fol}(c_3, c_1)))$, which can be rewritten as $E_1^n \models \mathtt {fol}(c_2, \mathtt {and}(c_1, c_3))$, which includes $E_1^n \models \mathtt {and}(c_1, c_3)$. $\square$

##### Proof

*Non-transitivity of the* $\mathtt {or}$ *operator* If $E_1^n \models \mathtt {or}(c_1, c_2)$, and $E_1^n \models \mathtt {or}(c_2, c_3)$, both formulas can be satisfied by matching the $c_2$ pattern only, and consequently, $E_1^n \models \mathtt {or}(c_1, c_3)$ does not hold necessarily. $\square$

### Appendix 2: Source Codes of the Gesture Recognition Case Study

See Figs. 18, 19, 20.

## Footnotes

1 In this paper, we do not investigate the hypothetical special case of two events having the same timestamp. In this case, two events are ordered randomly.

2
https://www.eclipse.org/viatra/.

3 The detailed description of the domains with advanced scenarios and sources is available from https://github.com/IncQueryLabs/incquery-examples-cps.

4 The complete source code is available from https://github.com/FTSRG/viatra-cep-examples/wiki/Complex-event-driven-quick-fix-generation.

5
https://www.eclipse.org/viatra/.

6 The complete source code is available from https://github.com/FTSRG/viatra-cep-examples/wiki/Streaming-model-transformations-over-Jnect.

## References

[1] Abdeen, H., Varró, D., Sahraoui, H., Nagy, A.S., Hegedüs, Á., Horváth, Á., Debreceni, C.: Multi-objective optimization in rule-based design space exploration. In: 29th IEEE/ACM International Conference on Automated Software Engineering (ASE 2014), pp. 289–300. IEEE, Vasteras, Sweden (2014). doi:10.1145/2642937.2643005

[2] Agrawal, J., Diao, Y., Gyllstrom, D., Immerman, N.: Efficient pattern matching over event streams. In: Proceedings of the 2008 ACM SIGMOD International Conference on Management of Data, SIGMOD ’08, pp. 147–160. ACM, New York, NY, USA (2008). doi:10.1145/1376616.1376634

[3] Alevizos, E., Artikis, A.: Being logical or going with the flow? A comparison of complex event processing systems. In: Likas, A., Blekas, K., Kalles, D. (eds.) Artificial Intelligence: Methods and Applications. Lecture Notes in Computer Science, vol. 8445. Springer International Publishing (2014). doi:10.1007/978-3-319-07064-3_40

[4] Allen JF (1983). Maintaining knowledge about temporal intervals. Commun. ACM.

[5] Apache Software Foundation: Apache Camel Official Website. http://camel.apache.org/. Accessed 24 April 2016

[6] Apache Software Foundation: Apache Camel RX Official Website. http://camel.apache.org/rx.html. Accessed 24 April 2016

[7] Arasu, A., Cherniack, M., Galvez, E., Maier, D., Maskey, A.S., Ryvkina, E., Stonebraker, M., Tibbetts, R.: Linear road: a stream data management benchmark. In: Proceedings of the Thirtieth International Conference on Very Large Data Bases, vol. 30, VLDB ’04, pp. 480–491. VLDB Endowment (2004). http://dl.acm.org/citation.cfm?id=1316689.1316732

[8] Asplund, F., Biehl, M., El-Khoury, J., Törngren, M.: Tool integration beyond wasserman. In: Salinesi, C., Pastor, O. (eds.) Advanced Information Systems Engineering Workshops. Lecture Notes in Business Information Processing, vol. 83, pp. 270–281. Springer Berlin Heidelberg (2011). doi:10.1007/978-3-642-22056-2_29

[9] AtlanMod: Reactive-ATL Website. http://web.emn.fr/x-info/atlanmod/index.php?title=Reactive-ATL. Accessed 24 April 2016

[10] Baader, F., Snyder, W., Narendran, P., Schmidt-Schauss, M., Schulz, K.: Chapter 8: Unification theory. In: Robinson, A., Voronkov, A. (eds.) Handbook of Automated Reasoning, Handbook of Automated Reasoning, pp. 445 – 533. North-Holland, Amsterdam (2001). doi:10.1016/B978-044450813-3/50010-2. http://www.sciencedirect.com/science/article/pii/B9780444508133500102

[11] Balogh, L., Dávid, I., Ráth, I., Varró, D., Vörös, A.: Distributed and heterogeneous event-based monitoring in smart cyber-physical systems. http://msdl.cs.mcgill.ca/people/istvan/pub/mtcps2016. Accessed 24 April 2016

[12] Bergmann, G., Boronat, A., Heckel, R., Torrini, P., Ráth, I., Varró, D.: Rigorous software engineering for service-oriented systems: results of the SENSORIA project on software engineering for service-oriented computing. In: Wirsing, M., Hölzl, M. (eds.) Advances in Model Transformations by Graph Transformation: Specification, Execution and Analysis. Springer, Heidelberg (2010)

[13] Bergmann, G., Dávid, I., Hegedüs, A., Horváth, A., Ráth, I., Ujhelyi, Z., Varró, D.: VIATRA 3: a reactive model transformation platform. In: Theory and Practice of Model Transformations. Lecture Notes in Computer Science. Springer, Berlin/Heidelberg (2015)

[14] Bergmann G, Ráth I, Varró G, Varró D (2012). Change-driven model transformations. Change (in) the rule to rule the change. Softw. Syst. Model..

[15] Blair G, Bencomo N, France R (2009). Models@run.time. Computer.

[16] Box, G., Jenkins, G., Reinsel, G.: Time Series Analysis: Forecasting and Control. Wiley Series in Probability and Statistics. Wiley (2008). http://books.google.hu/books?id=lJnnPQAACAAJ

[17] Brech, B., Jamison, J., Shao, L., Wightwick, G.: The Interconnecting of Everything. IBM Redbook (2013)

[18] Breu R, Agreiter B, Farwick M, Felderer M, Hafner M, Innerhofer-Oberperfler F (2011). Living models-ten principles for change-driven software engineering. Int. J. Softw. Inform..

[19] Bürger, C., Mey, J., Schöne, R., Karol, S., Langner, D.: Using reference attribute grammar-controlled rewriting for energy auto-tuning. In: 10th International Workshop on Models@ run. time. CEUR Workshop Proceedings (CEUR-WS. org) (2015)

[20] Cariou, E., Barbier, F., Goaer, O.L.: Model execution adaptation? In: Proceedings of the 7th Workshop on Models@run.time, MRT ’12, pp. 60–65. ACM, New York, NY, USA (2012). doi:10.1145/2422518.2422528

[21] Carlson, J., Lisper, B.: A resource-efficient event algebra. Sci. Comput. Program. **75**(12), 1215 – 1234 (2010). doi:10.1016/j.scico.2010.06.010. http://www.sciencedirect.com/science/article/pii/S016764231000122X

[22] Combemale, B., Thirioux, X., Baudry, B.: Formally defining and iterating infinite models. In: France, R. B., Kazmeier, J., Breu, R., Atkinson, C. (eds.) Model Driven Engineering Languages and Systems—15th International Conference, MODELS 2012, Innsbruck, Austria, September 30-October 5, 2012. Proceedings, LNCS, vol. 7590, pp. 119–133. Springer (2012)

[23] Cugola G, Margara A (2012). Complex Event Processing with T-REX. J. Syst. Softw..

[24] Cugola G, Margara A (2012). Processing flows of information: from data stream to complex event processing. ACM Comput. Surv..

[25] Dávid, I.: A model-driven approach for processing complex events. CoRR **abs/1204.2203** (2012). Accessed 24 April 2016

[26] Dávid, I., Ráth, I.: Realtime gesture recognition with Jnect and Esper. In: Tech demo at EclipseCon Europe 2012. http://incquery.net/incquery/demos/jnect. Accessed 23 June 2014

[27] Dávid, I., Ráth, I., Varró, D.: Streaming model transformations by complex event processing. In: Dingel, J., Schulte, W., Ramos, I., Abrahão, S., Insfran, E. (eds.) Model-Driven Engineering Languages and Systems. Lecture Notes in Computer Science, vol. 8767, pp. 68–83. Springer International Publishing (2014). doi:10.1007/978-3-319-11653-2_5

[28] Demers, A., Gehrke, J., Panda B.: Cayuga: a general purpose event monitoring system. In: CIDR, pp. 412–422 (2007)

[29] Derler P, Lee E, Vincentelli A (2012). Modeling cyber–physical systems. Proc. IEEE.

[30] Deshayes, R., Mens, T.: Statechart modelling of interactive gesture-based applications. In: Proceedings of First International Workshop on Combining Design and Engineering of Interactive Systems through Models and Tools (ComDeis-Moto), Lisbon, Portugal (2011), iNTERACT (2011)

[31] Dummit DS, Foote RM (2003). Abstract algebra.

[32] Eclipse Foundation: Eclipse Modeling Framework Project (EMF). http://www.eclipse.org/modeling/emf/. Accessed 24 April 2016

[33] Eclipse Foundation: Xbase Documentation. https://www.eclipse.org/Xtext/documentation/305_xbase.html#xbase-language-ref-introduction. Accessed 24 April 2016

[34] EsperTech Inc.: Esper Official Website. http://www.espertech.com/esper. Accessed 24 April 2016

[35] EsperTech Inc.: Performance-Related Information. http://www.espertech.com/esper/performance.php. Accessed 24 April 2016

[36] Esteves Veríssimo, P., Gönczy, L., Csertán, G., Urbanics, G., Ghani, H., Khelil, A., Suri, N.: Monitoring and evaluation of semantic rooms. In: Baldoni, R., Chockler, G. (eds.) Collaborative Financial Infrastructure Protection, pp. 99–116. Springer, Berlin (2012). doi:10.1007/978-3-642-20420-3_5

[37] Etzion O, Niblett P (2010). Event Processing in Action.

[38] Fontenla-Romero, Ó., Guijarro-Berdiñas, B., Martinez-Rego, D., Pérez-Sánchez, B., Peteiro-Barral, D.: Online machine learning. In: Igelnik, B., Zurada, J.M. (eds.) Efficiency and Scalability Methods for Computational Intellect, pp. 27–54. IGI Publishing Hershey, PA, USA (2013)

[39] Forgy, C.L.: Expert systems. In: RETE: a fast algorithm for the many pattern/many object pattern match problem, pp. 324–341. In: IEEE Computer Society Press, Los Alamitos, CA, USA (1990). http://dl.acm.org/citation.cfm?id=115710.115736

[40] Fusco, M., Sottara, D., Ráth, I., Proctor, M.: Building a hybrid reactive rule engine for relational and graph reasoning. In: Bassiliades, N., Gottlob, G., Sadri, F., Paschke, A., Roman, D. (eds.) Rule Technologies: Foundations, Tools, and Applications. Lecture Notes in Computer Science, vol. 9202, pp. 208–222. Springer International Publishing (2015). doi:10.1007/978-3-319-21542-6_14

[41] Hartmann, T., Moawad, A., Fouquet, F., Nain, G., Klein, J., Le Traon, Y.: Stream my models: reactive peer-to-peer distributed models@ run. time. In: 2015 ACM/IEEE 18th International Conference on Model Driven Engineering Languages and Systems (MODELS), pp. 80–89. Conference Publishing Consulting (2015)

[42] Hegedüs, Á., Horváth, Á., Ráth, I., Branco, M.C., Varró, D.: Quick fix generation for DSMLs. In: 2011 IEEE Symposium on Visual Languages and Human-Centric Computing, VL/HCC 2011, Pittsburgh, PA, USA, September 18–22, 2011, pp. 17–24 (2011). doi:10.1109/VLHCC.2011.6070373

[43] Hegedüs, Á., Horváth, Á., Varró, D.: A model-driven framework for guided design space exploration. Automated Software Engineering pp. 1–38 (2014). doi:10.1007/s10515-014-0163-1. http://link.springer.com/article/10.1007%2Fs10515-014-0163-1

[44] Helming, J., Neufeld, E., Koegel, M.: Jnect—An Eclipse Plug—In providing a Java Adapter for the Microsoft Kinect SDK. http://code.google.com/a/eclipselabs.org/p/jnect/. Accessed 24 April 2016

[45] Herzig, S.J., Qamar, A., Reichwein, A., Paredis, C.J.: A conceptual framework for consistency management in model-based systems engineering. In: ASME 2011 International Design Engineering Technical Conferences and Computers and Information in Engineering Conference, pp. 1329–1339. American Society of Mechanical Engineers (2011)

[46] Hinze, A., Voisard, A.: EVA: an event algebra supporting complex event specification. Inf. Syst. **48**(0), 1–25 (2015). doi:10.1016/j.is.2014.07.003. http://www.sciencedirect.com/science/article/pii/S0306437914001252

[47] Hopcroft JE, Motwani R, Ullman JD (2006). Introduction to Automata Theory, Languages, and Computation.

[48] Huang, J.: Performance measures of machine learning. Ph.D. thesis, Ont., Canada, Canada (2006). AAINR30363

[49] Iason Oikonomidis, N.K., Argyros, A.: Efficient model-based 3D tracking of hand articulations using Kinect. In: Proceedings of the British Machine Vision Conference, pp. 101.1–101.11. BMVA Press (2011). doi:10.5244/C.25.101

[50] IncQuery Labs: EMF-IncQuery CPS Demonstrator Wiki. https://github.com/IncQueryLabs/incquery-examples-cps/wiki. Accessed 24 April 2016

[51] IncQuery Labs: EMF-IncQuery Validation Framework Documentation. https://wiki.eclipse.org/VIATRA/Addon/Validation. Accessed 24 April 2016

[52] Jayasekara, S., Kannangara, S., Dahanayakage, T., Ranawaka, I., Perera, S., Nanayakkara, V.: Wihidum: distributed complex event processing. J. Parallel Distrib. Comput. (2015). doi:10.1016/j.jpdc.2015.03.002. http://www.sciencedirect.com/science/article/pii/S0743731515000519

[53] Kelly S, Tolvanen JP (2008). Domain-specific modeling: enabling full code generation.

[54] Kevoree Project: Kevoree Modeling Framework (KMF). http://kevoree.org/kmf/. Accessed 24 April 2016

[55] Kolovos, D.S., Ruscio, D.D., Matragkas, N.D., Cuadrado, J.S., Ráth, I., Tisi, M. (eds.): Proceedings of the 3rd Workshop on Scalable Model Driven Engineering part of the Software Technologies: Applications and Foundations (STAF 2015) federation of conferences, L’Aquila, Italy, July 23, 2015. CEUR Workshop Proceedings, vol. 1406. CEUR-WS.org (2015). http://ceur-ws.org/Vol-1406

[56] Lee, E.: Cyber physical systems: design challenges. In: 11th IEEE International Symposium on Object Oriented Real-Time Distributed Computing (ISORC), 2008 , pp. 363–369. IEEE (2008)

[57] Lee, E.A., Rabaey, J., Blaauw, D., Fu, K., Guestrin, C., Hartmann, B., Jafari, R., Jones, D., Kubiatowicz, J., Kumar, V., Mangharam, R., Murray, R., Pappas, G., Pister, K., Rowe, A., Sangiovanni-Vincentelli, A., Seshia, S.A., Rosing, T.S., Taskar, B., Wawrzynek, J., Wessel, D.: The swarm at the edge of the cloud. IEEE Des Test. **31**(3), 1–13 (2014). http://chess.eecs.berkeley.edu/pubs/1066.html

[58] Luckham DC (2001). The Power of Events: An Introduction to Complex Event Processing in Distributed Enterprise Systems.

[59] Microsoft Corp.: Microsoft Kinect official website. http://www.microsoft.com/en-us/kinectforwindows/. Accessed 24 April 2016

[60] Moawad, A., Hartmann, T., Fouquet, F., Nain, G., Klein, J., Traon, Y.L.: Beyond discrete modeling: a continuous and efficient model for iot. In: 18th ACM/IEEE International Conference on Model Driven Engineering Languages and Systems, MoDELS 2015, Ottawa, ON, Canada, September 30–October 2, 2015, pp. 90–99 (2015). doi:10.1109/MODELS.2015.7338239

[61] Ráth, I., Bergmann, G., Ökrös, A., Varró, D.: Live model transformations driven by incremental pattern matching. In: Theory and Practice of Model Transformations. Lecture Notes in Computer Science, vol. 5063/2008, pp. 107–121. Springer, Berlin, Heidelberg (2008). doi:10.1007/978-3-540-69927-9_8. http://www.springerlink.com/content/g43052uj0p27428v/

[62] Ráth, I., Horváth, A.: IoT Supercharged: Complex Event Processing for MQTT with Eclipse Technologies—Presentation. http://www.slideshare.net/IstvanRath (2015). Accessed 24 April 2016

[63] Red Hat Inc.: Drools Official Website. http://drools.org. Accessed 24 April 2016

[64] Saleh, O., Betz, H., Sattler, K.U.: Partitioning for scalable complex event processing on data streams. In: Bassiliades, N., Ivanovic, M., Kon-Popovska, M., Manolopoulos, Y., Palpanas, T., Trajcevski, G., Vakali, A. (eds.) New Trends in Database and Information Systems II, Advances in Intelligent Systems and Computing, vol. 312, pp. 185–197. Springer International Publishing (2015). doi:10.1007/978-3-319-10518-5_15

[65] Sánchez Cuadrado, J., de Lara, J.: Streaming model transformations: scenarios, challenges and initial solutions. In: Duddy, K., Kappel, G. (eds.) Theory and Practice of Model Transformations. Lecture Notes in Computer Science, vol. 7909, pp. 1–16. Springer, Berlin, Heidelberg (2013). doi:10.1007/978-3-642-38883-5_1

[66] Schmidt, K.U., Stühmer, R., Stojanovic, L.: Blending complex event processing with the RETE algorithm. In: Anicic, D., Brelage, C., Etzion, O., Stojanovic, N., (eds.) Proceedings of iCEP2008: 1st International Workshop on Complex Event Processing for the Future Internet, vol. 412. CEUR Workshop proceedings (2008)

[67] Schultz-Møller, N.P., Migliavacca, M., Pietzuch, P.: Distributed complex event processing with query rewriting. In: Proceedings of the Third ACM International Conference on Distributed Event-Based Systems, DEBS ’09, pp. 4:1–4:12. ACM, New York, NY, USA (2009). doi:10.1145/1619258.1619264

[68] Song, H., Huang, G., Chauvel, F., Zhang, W., Sun, Y., Shao, W., Mei, H.: Instant and incremental QVT transformation for runtime models. In: Proceedings of the 14th International Conference on Model Driven Engineering Languages and Systems. MODELS’11, pp. 273–288. Springer, Berlin, Heidelberg (2011)

[69] Szárnyas, G., Izsó, B., Ráth, I., Harmath, D., Bergmann, G., Varró, D.: IncQuery-D: a distributed incremental model query framework in the cloud. In: ACM/IEEE 17th International Conference on Model Driven Engineering Languages and Systems, MODELS 2014. Springer, Springer, Valencia, Spain (2014). Acceptance rate: 26 %

[70] Ujhelyi, Z., Bergmann, G., Hegedüs, Á., Horváth, Á., Izsó, B., Szatmári, Z., Varró, D.: An Integrated Development Environment for Live Model Queries. Sci. Comput. Program. **98**(1), 70–108 (2015)

[71] Vangheluwe, H.L.: DEVS as a common denominator for multi-formalism hybrid systems modelling. In: IEEE International Symposium on Computer-Aided Control System Design, 2000. CACSD 2000, pp. 129–134. IEEE (2000)

[72] Wasserman, A.: Tool integration in software engineering environments. In: Long, F. (eds.) Software Engineering Environments. Lecture Notes in Computer Science, vol. 467, pp. 137–149. Springer, Berlin, Heidelberg (1990). doi:10.1007/3-540-53452-0_38

[73] Weber RH, Weber R (2010). Internet of Things.

[74] Winkelmann J, Taentzer G, Ehrig K, Küster JM (2008). Translation of restricted OCL constraints into graph constraints for generating meta model instances by graph grammars. Electron. Notes Theor. Comput. Sci..

[75] Xia, L., Chen, C.C., Aggarwal, J.: Human detection using depth information by kinect. In: IEEE Computer Society Conference on Computer Vision and Pattern Recognition Workshops (CVPRW), 2011, pp. 15–22 (2011). doi:10.1109/CVPRW.2011.5981811

[76] Yskout, K., Scandariato, R., Joosen, W.: Change patterns: co-evolving requirements and architecture. Softw. Syst. Model. (2012). https://lirias.kuleuven.be/handle/123456789/334610

